# Development and Application of a Water Temperature Related Physiologically Based Pharmacokinetic Model for Enrofloxacin and Its Metabolite Ciprofloxacin in Rainbow Trout

**DOI:** 10.3389/fvets.2020.608348

**Published:** 2021-01-25

**Authors:** Fan Yang, Fang Yang, Dan Wang, Chao-Shuo Zhang, Han Wang, Zhe-Wen Song, Hao-Tian Shao, Mei Zhang, Meng-Li Yu, Yang Zheng

**Affiliations:** ^1^College of Animal Science and Technology, Henan University of Science and Technology, Luoyang, China; ^2^Environmental and Animal Products Safety Laboratory of Key Discipline in University of Henan Province, Henan University of Science and Technology, Luoyang, China; ^3^Jiaozuo Livestock Product Quality and Safety Monitoring Center, Jiaozuo, China

**Keywords:** enrofloxacin, ciprofloxacin, rainbow trout, physiologically based pharmacokinetic model, water temperature, residue

## Abstract

Enrofloxacin (ENR) has been approved for the treatment of infections in aquaculture, but it may cause tissue residue. This research aimed to develop and validate a water temperature related PBPK model, including both ENR and ciprofloxacin (CIP), in rainbow trout, and to predict further their residue concentrations and the withdrawal periods for ENR at different water temperatures. With the published concentrations data, a flow-limited PBPK model including both ENR and CIP sub-models was developed to predict ENR and CIP concentrations in tissues and plasma/serum after intravenous, oral, or immersion administration. A Monte Carlo simulation including 500 iterations was further incorporated into this model. Based on the model and Monte Carlo analysis, the withdrawal intervals were estimated for different dosage regimens and at different water temperatures, ranging from 80 to 272 degree-days. All of these values were shorter than the labeled withdrawal period (500 degree-days) in fish. This model provided a useful tool for predicting the tissue residues of ENR and CIP in rainbow trout under different dosage regimens and at different water temperatures.

## Introduction

World production of farmed aquatic animals is still the fastest-growing animal food-producing sector, which accounted for approximately 46% of the total fish source food supply (1). Rainbow trout is a minor species but with a relatively high worldwide consumption (2). In North China, turbot has become a vital aquaculture species (3). However, because of the high rearing density, fish are suffering from some severe infections by some pathogens, such as *Aeromonas hydrophila, Vibrio anguillarum, Aeromonas salmonicida, Lactococcus garvieae, Pseudomonas spp., Flavobacterium psychrophilum*, and *Yersinia ruckeri* (4–8). The infections may lead to severe mortality among trout and substantial financial losses for the trout industry. It is of great importance to treat infections with effective antibacterials.

Enrofloxacin (ENR) is a synthetic fluoroquinolone developed especially for veterinary application, and it acts by inhibition of bacterial DNA-gyrase (9). ENR has been demonstrated effective against all common aquatic pathogens mentioned above (10–13). It can be *in vivo* biotransformed to its metabolite ciprofloxacin (CIP) (13, 14), which has been extensively used in the human clinic for the last two decades. In China, ENR has been licensed for aquatic medical use, and its recommended doses were 10 to 20 mg/kg B.W. per day for 5 to 7 days, administered through mixing with the formulated feed (15). The official withdrawal period for ENR in aquaculture was 500 degree-days (15). In some other countries, bath dosing was also approved for ENR in aquaculture; however, the recommended doses varied among different counties (16, 17). In the United States (US) and the European Union (EU), the unregistered compounds (such as ENR) were approved to treat fish diseases in an extra-label manner. Under these circumstances, a standard withdrawal period (500 degree-days) has been imposed for these off-label compounds (18). Unlike the withdrawal periods in mammals, those in fish will be significantly affected by the water temperature because fish are heterothermic animals (19). Therefore, the drug withdrawal periods will dramatically vary at different water temperatures even under the same dosing schedule in fish.

In China, marker residue for ENR is the sum of ENR and CIP with a maximum residue limit (MRL) of 100 μg/kg in muscle plus skin (15). However, the official withdrawal period was not available for ENR in aquaculture animals in China. To our knowledge, the residue of ENR and its metabolite CIP in edible tissues from rainbow trout is relatively typical compared with the other antibacterials (16, 20). In Iran, the total concentration of ENR and CIP in rainbow trout edible tissue exceeded the MRL (100 μg/kg) by at least 18.92% of the collected sample (*n* = 74) (20). Similar results have also been found in other countries (16). Excessive residues of antibiotics in fish tissues may lead to adverse effects on human health (21). Allergic reactions, gastrointestinal disturbances, carcinogenic effects, and photosensitivity constituted direct toxic effects (16). In addition to the direct toxic effects, consumers are more worried about the prevalence of antibiotic resistance through selective pressure on bacteria (22). A study conducted in rats has indicated that ENR had some acute toxic effects, including reducing body weight gain, caecal distension, degenerative changes in the knee joint, and the testicular effects in male subjects (23). These results showed that it is essential to monitor the residues of ENR and related compounds in foods of animal origin.

There have been increasing studies on predicting veterinary drug residues (24–27) and drug withdrawal periods (21, 28–31) based on the PBPK model. The PBPK model is based on the mass balance equation and allows the application of population variability data and *in vitro* mechanism to predict drug concentration in edible tissues (32). Some models have been developed in rainbow trout for environmental pollutants (33–36), rather than veterinary drugs. Also, neither of those previous models investigated the impact of water temperature on the compound disposition. Considering the wide range of climatic zones in which trout have been cultivated, it is important to develop a water temperature related PBPK model containing both ENR and its metabolite CIP to forecast tissue residues in trout. The objectives of the current study were (i) to establish and validate a water temperature related PBPK model containing both ENR and CIP in rainbow trout; (ii) to forecast the tissue residues and the withdrawal periods for ENR at different water temperatures based on this model.

## Materials and Methods

### Concentrations vs. Time Data of ENR and CIP

The published studies regarding ENR and rainbow trout have been widely searched, and the pharmacokinetics or depletion studies conducted in rainbow trout after intravenous (IV), oral (PO), or bath administration (IB) were chosen. The concentrations of ENR and CIP in plasma, serum, and tissue were read directly or extracted from the previously published figures using the software of GetData Graph Digitizer (version 2.26). The key information is given in Table 1 about those selected studies.

**Table 1:** Literature used in the model optimization and validation.

**Purpose^**a**^**	**Routes^**b**^**	**Dose (mg/kg)**	**BW (g)**	**Water temperature (°C)**	**Compounds**	**Matrix^**d**^**	**Ref**.
OP	IV	5, 10	100	15	ENR	Se	(37)
VA	PO	5, 10, 50		10, 15			
OP	PO	10	150 ± 5	10 ± 0.3, 16 ± 0.8	ENR	Pl, Mu, Li, Ki	(3)
OP				10 ± 0.3, 16 ± 0.8	CIP		
VA	IV			16 ± 0.8	ENR	Pl	
OP	IB	20 ^**c**^	204 ± 32	16.3 ± 0.3	ENR	Pl, Sk, Mu, Li, Ki, Gu	(38)
VA	IB	100 ^**c**^					
VA	PO	10					
OP	PO	10	450	17	ENR, CIP	Se, Li, Mu	(13)
VA	PO	30	50 ± 5	5 ± 0.5	ENR, CIP	Pl, Li, Mu	(39)
				10 ± 0.5			
				15 ± 0.5			

*BW, body weight*.

a *The abbreviations for the purpose: VA, Validation; OP, Optimization*.

b *The abbreviations for routes: IV, intravenous injection; PO, oral administration; IB, immersion bath*.

c *These doses are those for immersion bath administration, 20 represents immersion bath in the water with a concentration of ENR at 20 ppm for 2.5 hours, and 100 represents immersion bath in the water containing ENR at the concentration at 100 ppm for 0.5 hours*.

d *The abbreviations for matrix: Se, serum; Pl, plasma; Mu, muscle; Li, liver; Ki, kidney; Gu, gut; Sk, Skin*.

### Model Structure

**Figure 1:**
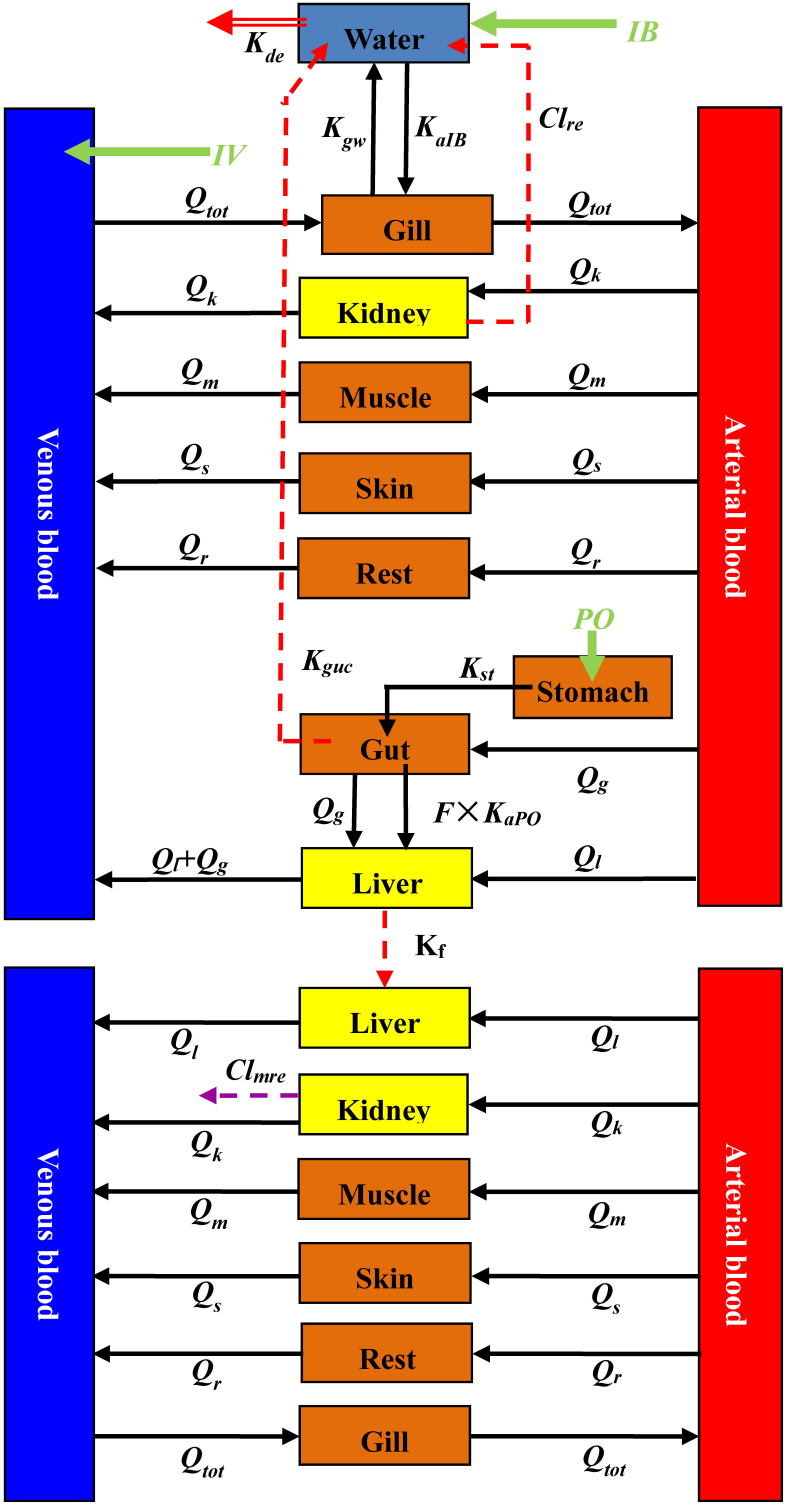
A schematic diagram of the current PBPK model for ENR and CIP in rainbow trout. Q_x_ (L/h) is blood flow through some tissue. A specific tissue was distinguished by its subscript x, and tissues of kidney, muscle, skin, gut, liver, and the rest of the body compartment were abbreviated as k, m, s, g, l, and r, respectively. Q_tot_ is the cardiac output, indicating that all cardiac output flows through the gill. Three dosing routes (IV, IB, and PO) were included in the model. The abbreviations of IV and PO are all ENR doses intravenously and orally administrated to rainbow trout, respectively. The symbol of IB represents total exposure through an immersion bath. After IV dosing, ENR enters into the venous blood completely and directly. Following PO administration, the drugs directly enter the stomach and then enter the small intestine under the action of gastric emptying, where some of them are absorbed into the blood circulation. Those unabsorbed drugs are eliminated from the fish body to the culture water. K_st_ (h^−1^) is the rate constant of gastric emptying, K_aPO_ (h^−1^) is the rate constant of absorption, and K_guc_ (h^−1^) is the rate constant of elimination with feces. F represents oral bioavailability. During the immersion bath, ENR is absorbed into the venous blood through the gill with the absorption rate constant of K_aIB_ (h^−1^). From gill to water, another distribution rate constant is K_gw_ (h^−1^). ENR circulates to various tissues with the blood and is eliminated by liver metabolism and renal excretion. K_f_ (h^−1^) is the metabolism rate constant, and Cl_re_ (L/h/kg) is the renal clearance of ENR. When CIP is bio-transformed from ENR, it also circulates to various tissues. Then it is further eliminated by renal excretion, and the renal clearance for CIP is Cl_mre_ (L/h/kg). ENR and CIP in the fish body are all excreted into the water; however, the CIP concentrations were not predicted because of their low levels. ENR in water has a degradation rate constant, K_de_ (h^−1^).

The current PBPK model contained ENR and its metabolite CIP sub-models connected through hepatic metabolism. The sub-model of ENR had 10 compartments, including the digestive tract (stomach and intestine), liver, kidney, muscle, skin, gill, venous blood, arterial blood, and a virtual compartment representing the rest of the body (Figure 1). Compared with the ENR sub-model, the stomach and intestine were not incorporated in the CIP sub-model (Figure 1). As PO and IB are the approved administration routes for ENR in rainbow trout, they were both included in the present ENR sub-model. Additionally, the IV injection was also incorporated into the ENR sub-model to determine the same distribution and elimination profiles under all three administration routes. A compartment representing culture water was applied to simulate drug absorption after IB administration. An enterohepatic circulation module for ENR was initially included in this model; however, that module could not increase the accuracy of the prediction and even reduced the prediction accuracy for ENR and CIP concentrations in the liver (data not shown here). Therefore, the it was not finally included in the present study. Based on the previous PBPK models for other quinolones (28, 29, 40), flow-limited compartments were used for both ENR and CIP in the present study. The present PBPK model was developed based on the software platform of acslXtreme (version 3.0.2.1). The entire code for this model is provided in the Supplementary Materials.

For the IV route, ENR was directly injected into venous blood at the IV dose (mg/kg b.w.) and the duration of the infusion period (timeiv) was set as 0.001 h (41). For the IB route, the initial concentration of ENR in the water was 20 and 100 ppm, and the exposure periods (timeib) were 2.5 and 0.5 h, respectively (38). During the immersion bath, the drug was assumed to be only absorbed from the gill to the blood circulation, with the absorption rate constant of K_aIB_ and a complete 100% absorption (Figure 1). For the PO route, a two-compartment model including stomach and gut was applied to simulate the absorption based on our previous study (26). It was assumed that ENR entered the stomach directly and then was transported into the gut through gastric emptying at the rate of K_st_. Once in the intestine, most ENR will be absorbed with a rate constant of K_aPO_, and the bioavailability was 66.13% (3). Those unabsorbed drugs will be excreted from the intestinal contents with the feces into the culture water (Figure 1). And the excretion rate was abbreviated as K_guc_.

Following IV injection or extravascular absorption, ENR was distributed through the bloodstream to all tissue compartments. As reported anywhere (26, 28, 29, 41), the mass balance equations were coded to describe the concentration or mass change for ENR or CIP in each compartment (Table 2). According to the previous report (23), ENR is mainly eliminated by liver metabolism and renal excretion. Therefore, parameters of K_f_ and Cl_re_ were applied to simulate both processes, respectively. K_f_ was the rate constant of biotransformation from ENR to CIP in liver and Cl_re_ was an abbreviation for ENR renal clearance. The drugs excreted by the kidneys directly entered the aquaculture water (Figure 1). Besides the intestinal and renal excretions, some ENR was directly excreted from the gill to the aquaculture water, and the excretion rate constant was K_gw_ (Figure 1).

**Table 2:** Differential equation describing the change rate of ENR and CIP concentrations (mol/L) or mass (mol) in each compartment.

**Compound**	**Compartment**	**Differential equations**
ENR	Water	$\frac{dA_{w}}{dt}=\frac{IB}{timeib}\text{-}K_{de}\times A_{w}+K_{guc}\times A_{ic}+K_{gw}\times A_{gi}-K_{aIB}\times A_{w}+Cl_{re}\times bw\times \frac{C_{k}}{P_{k}}$
	Gill	$V_{gi}\times \frac{dC_{gi}}{dt}=K_{aIB}\times A_{w}-K_{gw}\times A_{gi}+Q_{tot}\times \left(C_{vp}-\frac{C_{gi}}{P_{gi}}\right)$
	Liver	$V_{l}\times \frac{dC_{l}}{dt}=Q_{g}\times \frac{C_{g}}{P_{g}}+\left(Q_{l}-Q_{g}\right)\times C_{ap}+K_{aPO}\times F_{PO}\times A_{ic}\text{-}K_{f}\times A_{l}\text{-}Q_{l}\times \frac{C_{l}}{P_{l}}$
	Kidney	$V_{k}\times \frac{dC_{k}}{dt}=Q_{k}\times \left(C_{ap}-\frac{C_{k}}{P_{k}}\right)-Cl_{re}\times bw\times \frac{C_{k}}{P_{k}}$
	Muscle	$V_{m}\times \frac{dC_{m}}{dt}=Q_{m}\times \left(C_{ap}-\frac{C_{m}}{P_{m}}\right)$
	Skin	$V_{s}\times \frac{dC_{s}}{dt}=Q_{s}\times \left(C_{ap}-\frac{C_{s}}{P_{s}}\right)$
	Stomach contents	$\frac{dA_{stc}}{dt}=\frac{PO}{timepo}-K_{st}\times A_{stc}$
	Intestinal contents	$\frac{dA_{ic}}{dt}=K_{st}\times A_{stc}-\left(F_{PO}\times K_{aPO}+K_{guc}\right)\times A_{ic}$
	Gut	$V_{g}\times \frac{dC_{g}}{dt}=Q_{g}\times \left(C_{ap}-\frac{C_{g}}{P_{g}}\right)$
	Rest	$V_{r}\times \frac{dC_{r}}{dt}=Q_{r}\times \left(C_{ap}-\frac{C_{r}}{P_{r}}\right)$
	Venous blood	$V_{vp}\times \frac{dC_{vp}}{dt}=\frac{IV}{timeiv}+Q_{k}\times \frac{C_{k}}{P_{k}}+Q_{l}\times \frac{C_{l}}{P_{l}}+Q_{m}\times \frac{C_{m}}{P_{m}}+Q_{s}\times \frac{C_{s}}{P_{s}}+Q_{r}\times \frac{C_{r}}{P_{r}}-Q_{tot}\times C_{vp}$
	Arterial blood	$V_{ap}\times \frac{dC_{ap}}{dt}=Q_{tot}\times \left(\frac{C_{gi}}{P_{gi}}-C_{ap}\right)$
CIP	Gill	$V_{gi}\times \frac{dC_{mgi}}{dt}=Q_{tot}\times \left(C_{mvp}-\frac{C_{mgi}}{P_{mgi}}\right)$
	Liver	$V_{l}\times \frac{dC_{ml}}{dt}=K_{f}\times A_{l}+Q_{l}\times \left(C_{map}-\frac{C_{ml}}{P_{ml}}\right)$
	Kidney	$V_{k}\times \frac{dC_{mk}}{dt}=Q_{k}\times \left(C_{map}-\frac{C_{mk}}{P_{mk}}\right)-Cl_{mre}\times bw\times \frac{C_{mk}}{P_{mk}}$
	Muscle	$V_{m}\times \frac{dC_{mm}}{dt}=Q_{m}\times \left(C_{map}-\frac{C_{mm}}{P_{mm}}\right)$
	Skin	$V_{s}\times \frac{dC_{ms}}{dt}=Q_{s}\times \left(C_{map}-\frac{C_{ms}}{P_{ms}}\right)$
	Rest	$V_{r}\times \frac{dC_{mr}}{dt}=Q_{r}\times \left(C_{map}-\frac{C_{mr}}{P_{mr}}\right)$
	Venous blood	$V_{vp}\times \frac{dC_{mvp}}{dt}=Q_{k}\times \frac{C_{mk}}{P_{mk}}+Q_{l}\times \frac{C_{ml}}{P_{ml}}+Q_{m}\times \frac{C_{mm}}{P_{mm}}+Q_{s}\times \frac{C_{ms}}{P_{ms}}+Q_{r}\times \frac{C_{r}}{P_{r}}-Q_{tot}\times C_{mvp}$
	Arterial blood	$V_{ap}\times \frac{dC_{map}}{dt}=Q_{tot}\times \left(\frac{C_{mgi}}{P_{mgi}}-C_{map}\right)$

*A_x_ and C_x_ are the amount (mol) and concentration (mol/L) of ENR in each compartment, respectively, while C_mx_ is the concentration (mol/L) of CIP. V_x_ and Q_x_ are volume (L) of and blood flow (L/h) through a tissue, respectively, while P_x_ and P_mx_ are the partition coefficients (unitless) for ENR and CIP, respectively. Subscript x is the name of a compartment, and w, stc, ic, gi, vp, ap, l, g, k, m, s, and r are abbreviations for the water for fish culturing, stomach contents, intestinal contents, gill, venous blood, arterial blood, liver, gut, kidney, muscle, skin, and the rest of the body compartment, respectively. Q_tot_ (L/h) is the cardiac output, indicating that all cardiac output flows through the gill. The symbols of PO, IV, and IB represent an oral, intravenous, and immersion bath dose of ENR (mol; more details about the translation of unit from mg/kg BW or ppm in water to mol can be found in the model code), respectively. Symbol of timepo and timeib represent the length of oral administration and immersion bath, respectively. The former value was set as 0.001 h in the present model, and the later was set to be the real values according to a previous report (38). The symbol of timeiv represents intravenous infusion time (0.001 h). The BW is abbreviated for body weight (kg), and t is for time (hour). Cl_re_ and Cl_mre_ are the renal clearances (L/h/kg) of ENR and CIP, respectively, which are both normalized by body weight. K_st_ (h^−1^) is the rate constant of gastric emptying, K_aPO_ (h^−1^) is the oral absorption rate constant, and K_guc_ (h^−1^) is the elimination rate constant with feces for the unabsorbed ENR. F represents the oral bioavailability. K_aIB_ (h^−1^) is the absorption rate constant following the immersion bath, K_gw_ (h^−1^) represents the distribution rate constant for ENR from gills to water. K_f_ (h^−1^) is the metabolism rate constant from ENR to CIP. All other symbols not explained here can be found in Figure 1 or the model code*.

After biotransformation, CIP was gradually distributed to the other tissues through the bloodstream. Since the primary elimination route of CIP was via urine (23), CIP was assumed to be eliminated only through renal excretion, and the parameter of Cl_mre_ (abbreviation for renal clearance) was used to simulate this process. Because of the low levels of CIP in tissues and aquaculture water after ENR administration (38, 39), we did not simulate the CIP concentrations in the aquaculture water. More details on the equations simulating the ENR and CIP dispositions could be found in Table 2 and the model code presented in the Supplementary Materials.

### Model Parameterization

It is worth emphasizing that all common parameters included in different dosing route models, such as Cl_re_, Cl_mre_, K_f_, etc., shared their same values. Since both serum and plasma concentrations previously reported (Table 1) were used to optimize and validate the current model, the parameters of pre and hematocrit (pcv) were used to calculate the ratios of serum and plasma to the total volume of whole blood, respectively. The pre and pcv values were set as 55.5 and 30.4%, respectively (42). To simplify the current simulation, neither ENR nor CIP was assumed to enter blood cells, and the amount of each compound in blood was equal to the amount in plasma or serum.

The model parameters, including blood flow (Q_cx_), tissue weight (V_cx_), and the partition coefficients between tissue and plasma for both ENR and CIP (abbreviated as P_x_ and P_mx_, respectively), are presented in Table 3. The parameters for tissue weight were derived from previous PBPK models in rainbow trout (33), crucian carp (24), and grass carp (25). The blood flow parameters were also derived from previous PBPK models in fish (24, 33). Tissue weight and blood flow were expressed as fractions of body weight (b.w.) and total cardiac output, respectively (Table 3). Because the mean body weights were varied among the trout in the previous studies (Table 1), the current model was adjusted based on the specific b.w. reported in each study. According to a previous report (42), the water temperature significantly affected the cardiac output of rainbow trout. And the cardiac output was estimated based on the following linear equation between water temperature (Temp, °C) and total cardiac output (CO, ml/min/kg): CO= Temp × 3.95–12.9 (42). Partition coefficients for ENR were calculated according to the area method (43), based on the ENR concentrations in plasma and tissues derived from a previous report (38). Three dosage regimens were applied in that study. Therefore, we calculated the P_x_ values based on the average AUC values. It was assumed that the P_mx_ values in the tissues of muscle, skin, gut, liver, and kidney in rainbow trout were equal to those reported in humans (40). Those in the other compartments (gill and the rest compartment) were optimized through a maximum likelihood algorithm of Nelder-Mead based on the previously reported concentrations of CIP in serum, muscle, and liver (13). In addition to P_mgi_ and P_mr_, the values of P_gi_ and P_r_ were also obtained through parameter optimization. More details about the optimization process could be found below.

**Table 3:** Parameters of tissue weights, blood flows, and tissue/plasma partition coefficients used in this physiologically based pharmacokinetic model.

**Compartment**	**Tissue weight^**a**^ (V_**cx**_, a fraction of bodyweight)**	**Blood flow^**f**^ (Q_**cx**_, a fraction of cardiac output)**	**Partition coefficient for ENR^**h**^ (P_**x**_)**	**Partition coefficient for CIP^**k**^ (P_**mx**_)**
Gill	0.039^**b**^	1	3.46^**i**^	2.45^**i**^
Liver	0.0126^**c**^	0.029^**c**^	4.9	3.67
Kidney	0.00841^**c**^	0.056^**c**^	11.53	8.2
Muscle	0.66^**c**^	0.6^**c**^	2.83^**j**^	1.6
Skin	0.1^**b**^	0.053^**b**^	7.38	0.718
Gut	0.0852^**b**^	0.1539^**b**^	4.88	3.39
Arterial blood	0.015^**d**^	NA	NA	NA
Venous blood	0.059^**d**^	NA	NA	NA
Rest	0.02079^**e**^	0.1081^**g**^	0.13^**i**^	0.15^**i**^

a *Mean body weight (BW) was different in the published studies, so this model was adjusted according to the average BW of rainbow trout used in the previous study*.

b *These values were taken from a previously reported PBPK model for florfenicol in another fish species, crucian carp (24)*.

c *These values were taken from a previously reported PBPK model for paraoxon in rainbow trout (33)*.

d *These values were taken from a previously reported PBPK model for doxycycline in another fish species, grass carp (25)*.

e *This value was calculated as 1–(0.039 + 0.0126 + 0.00841 + 0.66 + 0.1 + 0.0852 + 0.015 + 0.059)*.

f *Cardiac output (CO) was estimated based on the linear equation between cardiac output (CO, ml/min/kg) and acclimation temperature (Temp, °C) (42)*.

g *This value was calculated as 1–(0.029 + 0.056 + 0.6 + 0.053 + 0.1539)*.

h *Partition coefficients for ENR were calculated according to the area method (43), based on the ENR concentrations in plasma and tissues (including muscle, skin, gut, kidney, and liver) derived from a previous report (38). Three dosage regimens were applied in that study, including one single oral dosing and two immersion baths with different drug concentrations. Therefore, we calculated the partition coefficient for ENR based on the average AUC values*.

i *These values were obtained through parameter optimization*.

j *This value was taken from a previous study (13)*.

k *Partition coefficients for CIP in the tissues of muscle, skin, gut, liver, and kidney in rainbow trout were assumed to be equal to those previously reported in humans (40). Those in the other compartments (gill and the rest of the body) were optimized using a maximum likelihood algorithm of Nelder-Mead based on the concentrations of CIP in serum, muscle, and liver (13)*.

In addition to some partition coefficients for ENR and CIP mentioned above, some other parameters were unavailable. Therefore, their values were also optimized based on the published ENR and CIP concentrations (Table 1). It should be noted that all optimizations were performed in acslXtreme through the Nelder-Mead algorithm. During optimization, those ENR concentrations in plasma after IV dosing (37) were initially applied to optimize the parameters included by three different routes of the model, including P_gi_, P_r_, K_f_, and Cl_re_. After obtaining final values of these parameters were, the plasma concentrations of ENR (Table 1) published by the others (3) were used to validate these parameter values through visual comparisons of the predictions and observations. If most predicted concentrations were consistent with those published ones, these optimized values were determined. Next, the other parameters only used in the PO model were optimized through the published ENR concentrations in plasma and tissues (3), including K_st_, K_aPO_, and K_guc_. The other concentration data reported in plasma, serum, or tissues (Table 1) were used to validate these optimized values. Like the PO model, the parameters of K_aIB_, K_gw_, and K_de_ used in the IB model were next optimized based on the published ENR concentrations (38). The other published concentrations (Table 1) were used for the validation of these optimizations.

When these parameters for ENR were determined, the published ENR and CIP concentrations following PO dosing at different water temperatures (Table 1) were applied to optimize the parameters for CIP used in all routes of the model, including P_mr_, P_mgi_, and Cl_mre_. And the other concentrations of ENR and CIP were utilized to verify these optimizations (Table 1). Till this moment, all the model parameters were determined and fixed. Please note that all simulations were adjusted according to the administration routes and dosage, fish b.w., and water temperatures used in those previous studies; additionally, each validation was carried out immediately after the corresponding optimization.

### Model Validation

The current model was validated by visual comparisons of the present predictions and the previous observations. It should be emphasized that all of those observations for validations were not used in the optimization process. Linear regression analysis with the determination coefficient was also performed to validate this model. According to a previous study (31), a determination coefficient >0.75 is regarded as a general criterion for good prediction. Also, the mean absolute percentage error (MAPE) was calculated to validate this model (41). The evaluation criteria of MAPE are: (i) excellent prediction: MAPE < 10%; (ii) good prediction: 10% < MAPE < 20%; and (iii) acceptable prediction: MAPE < 50% (26).

### Sensitivity Analysis

A sensitivity analysis was carried out according to the previous study (41). In the sensitivity analysis process, the central difference method was utilized; more details could be found in our previous studies (26, 41). If the absolute value of its NSC was >0.25 (41), this parameter was considered as an influential one. In order to simultaneously perform the sensitivity analysis on all parameters, three routes of administration were simulated simultaneously. The PO and IV doses were simulated at 10 mg/kg b.w., while for the IB route, the exposure concentrations and time in water were 10 ppm and 1 h, respectively.

### Monte Carlo Analysis

Up to 48 parameters were included in the current model. Due to the computational complexity, it was impossible to perform a Monte Carlo simulation for all parameters. Therefore, a Monte Carlo analysis was only performed on the influential parameters (see the above sensitivity analysis for details). Their central tendency and spread, expressed as mean and standard deviation respectively, were from the previous studies (3, 13, 24, 25, 37–40) or obtained by parameter optimization (see the model parameterization above for details). Because of the scarcity of SD values for some influential parameters, their SD values were set to be 10% of their mean values. Normal distribution was assumed for these parameters, and their average, standard deviation (SD), lower limit (Mean-SD), and upper limit (Mean + SD) are presented in Supplementary Table 1. Only ENR orally administered was approved for aquacultural application in China; therefore, single and multiple oral dosing were simulated by the Monte Carlo analysis. In addition to oral administration, IB dosing was also commonly applied in aquatic clinics. Therefore, a Monte Carlo analysis was also performed for IB exposure. In order to determine the temperature effects, three different water temperatures (5, 10, and 16°C) were simulated in each analysis. All these analyses were undertaken through the Monte Carlo wizard in the acslXtreme software. And each Monte Carlo analysis contained 500 iterations.

### Withdrawal Interval Estimation

A 500-iteration Monte Carlo analysis was included in the current model, and different dosage regimens were simulated (see the Monte Carlo analysis listed above). After every run, the predicted concentrations of ENR, CIP, and ENR plus CIP vs. time data were all automatically recorded by acslXtreme. When all 500 runs were finished, it was equivalent to obtaining the concentration-time data in 500 virtual individuals. These data were used to calculate the withdrawal interval in each individual further to ensure the predictions below the corresponding MRL for each individual (41). According to the guidance of CCVP (15), a 99th percentile was used for withdrawal interval calculation. The M-type code for withdrawal interval estimation is presented in the Supplementary Materials. The residue target tissue in fish approved for ENR in China was muscle and skin in natural proportions; however, a previous study indicated that the ENR residue seemed to be especially bound to fish skin (44). Therefore, we divided the skin and muscle into two separate compartments in the current model and calculated the withdrawal periods in both of them. More details about the withdrawal interval calculation can be found in the Supplementary Materials or our previous study (41).

## Results

### Model Parameters

The anatomical and physiological parameter values used here were derived from previous models (24, 25, 33) and are listed in Table 3. The partition coefficient for ENR were calculated based on the area method (43), and their final values were 4.9, 11.53, 2.83, 7.38, and 4.88 in the liver, kidney, muscle, skin, and gut, respectively. Partition coefficients for ENR in gills and the rest of the body were obtained by parameter optimization, and their final values were 3.46 and 0.13, respectively. Partition coefficient values for CIP were assumed to be equal to those previously used in humans (40), with their final values of 3.67, 8.2, 1.6, 0.718, and 3.39 in the liver, kidney, muscle, skin, and gut, respectively. Similar to P_gi_ and P_r_, the values of P_mgi_ and P_mr_ were also acquired by parameter optimization with the final values of 2.45 and 0.15, respectively. The parameters related to absorption after PO or IB administration were also determined through optimization, whose final values were 0.175 h^−1^, 0.052 h^−1^, 1.103 h^−1^ for K_st_, K_aPO_, and K_aIB_, respectively (Table 4). The parameters related to the eliminations of ENR or CIP were also obtained through optimization, whose values were 0.0725 h^−1^, 0.058 L/kg/h, 0.605 h^−1^, 0.061 h^−1^, 12003.310 h^−1^, and 116.14 L/kg/h for K_f_, Cl_re_, K_guc_, K_gw_, K_de_, and Cl_mre_, respectively.

**Table 4:** The results of parameters optimization used under all routes administration based on the published concentrations after intravenous injection.

**Optimization order**	**Routes**	**Concentrations used to optimize parameters**		**Parameters for ENR (unit)**	**Parameters for CIP (unit)**	**Initial value**	**Final value**	**Standard deviation**
		**Ref**.	**Data description**					
1	All	(37)	Plasma concentrations of ENR after a single IV dose of ENR given to rainbow trout reared at 15°C at 5 or 10 mg/kg BW	P_gi_ (unitless)		1	3.46	1.21
				P_r_ (unitless)		0.1	0.13	0.04
				K_f_ (1/h)		0.05	0.0725	0.02
				Cl_re_ (L/kg/h)		0.05	0.058	0.02
2	PO	(3)	Plasma and tissue concentrations of ENR after a single PO dose of ENR given to rainbow trout reared at 10 and 16°C at 10 mg/kg BW	K_st_ (1/h)		0.1	0.175	0.004
				K_aPO_ (1/h)		0.1	0.052	0.001
				K_guc_ (1/h)		0.2	0.605	0.014
3	IB	(38)	Plasma and tissue concentrations of ENR and CIP after an immersion bath in the water with the concentration of ENR at 100 ppm during 0.5 h	K_aIB_ (1/h)		0.1	1.103	0.112
				K_gw_ (1/h)		0.1	0.061	0.075
				K_de_ (1/h)		1.2	12003.310	59.474
4	PO	(13)	Serum and tissue concentrations of ENR and CIP after a single PO dose of ENR (10 mg/kg BW) given to rainbow trout reared at 17°C		P_mr_ (unitless)	0.1	0.15	0.07
					P_mgi_ (unitless)	0.1	2.45	0.56
		(3)	Plasma and tissue concentrations of CIP after a single PO dose of ENR given to rainbow trout reared at 10 and 16°C at 10 mg/kg BW					
					Cl_mre_ (L/kg/h)	1	116.14	23.21

### Model Validation

**Figure 2:**
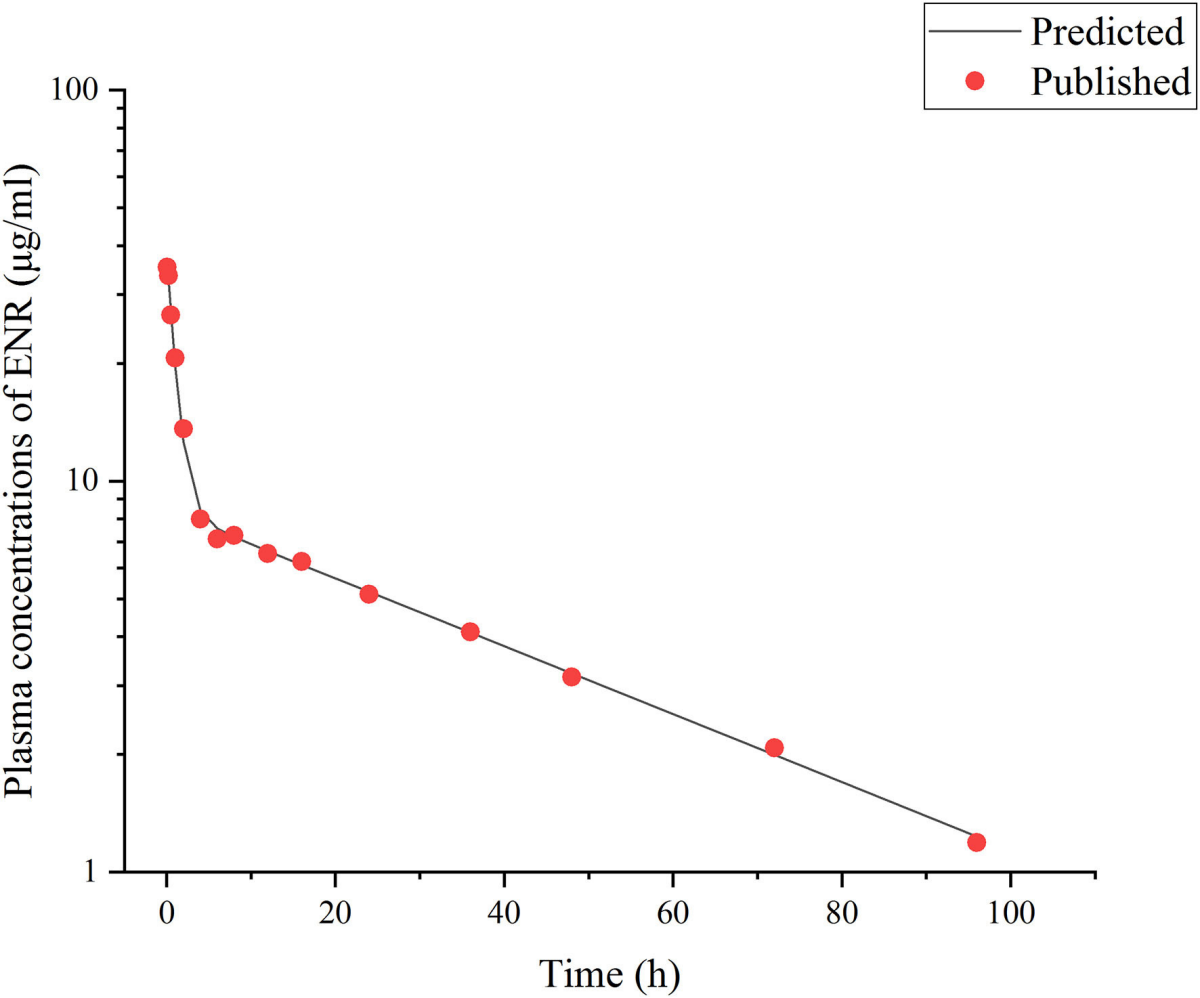
Comparisons between the predicted (curves) and published [points; (3)] ENR concentrations (μg/ml) in plasma after a single IV dose at 10 mg/kg b.w. in rainbow trout reared at the water temperature of 16°C.

**Figure 3:**
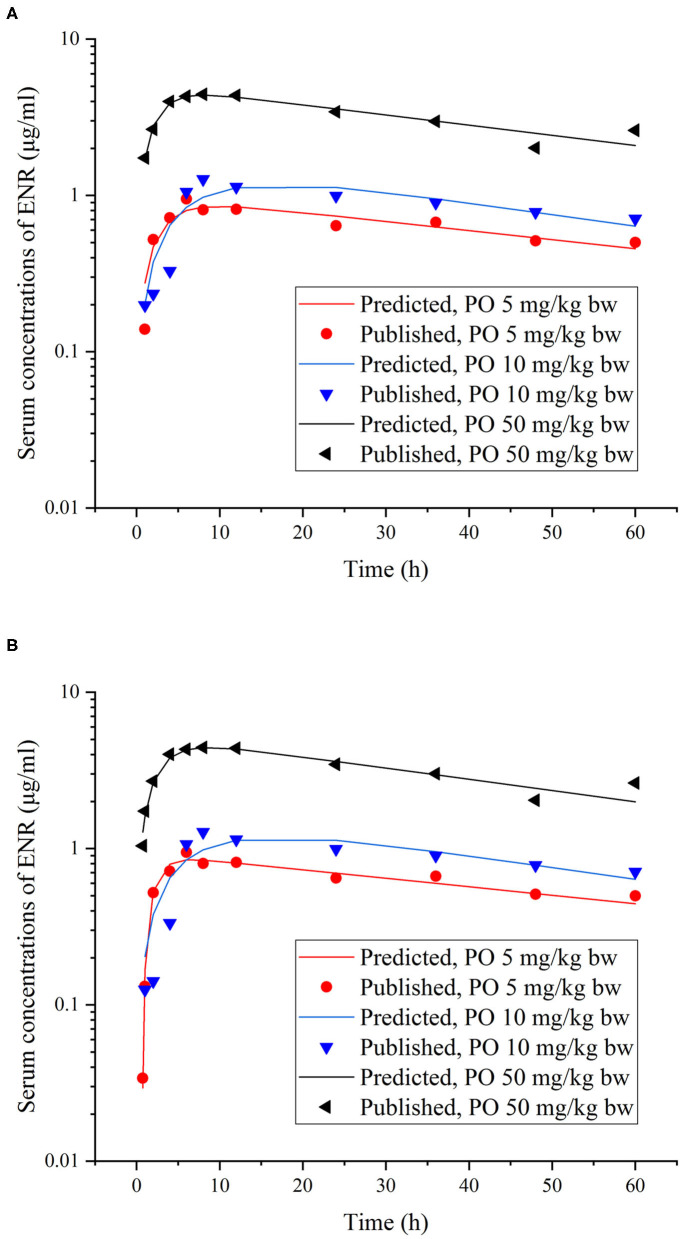
Comparisons between the predicted (curves) and published [points; (37)] ENR concentrations (μg/ml) in serum after different single PO doses in rainbow trout reared at two different water temperatures [**(A)**, at 10°C; **(B)**, at 15°C].

**Figure 4:**
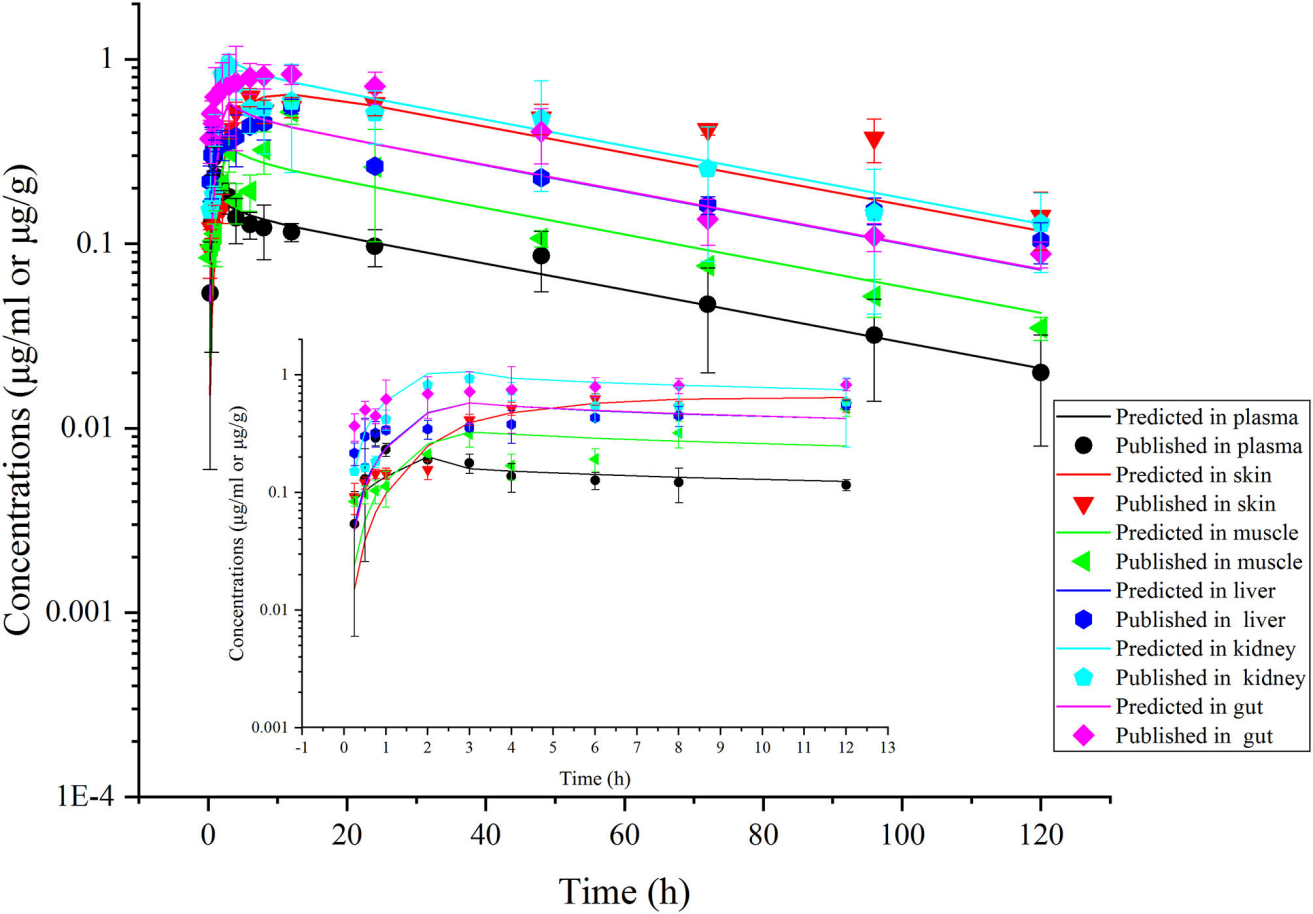
Comparisons between the predicted (curves) and published [points; (38)] ENR concentrations (μg/ml or μg/g) in trout plasma and tissues after an immersion bath in water with ENR concentration at 20 ppm for 2.5 h. The rainbow trout were reared at the water temperature of 16.3°C.

**Figure 5:**
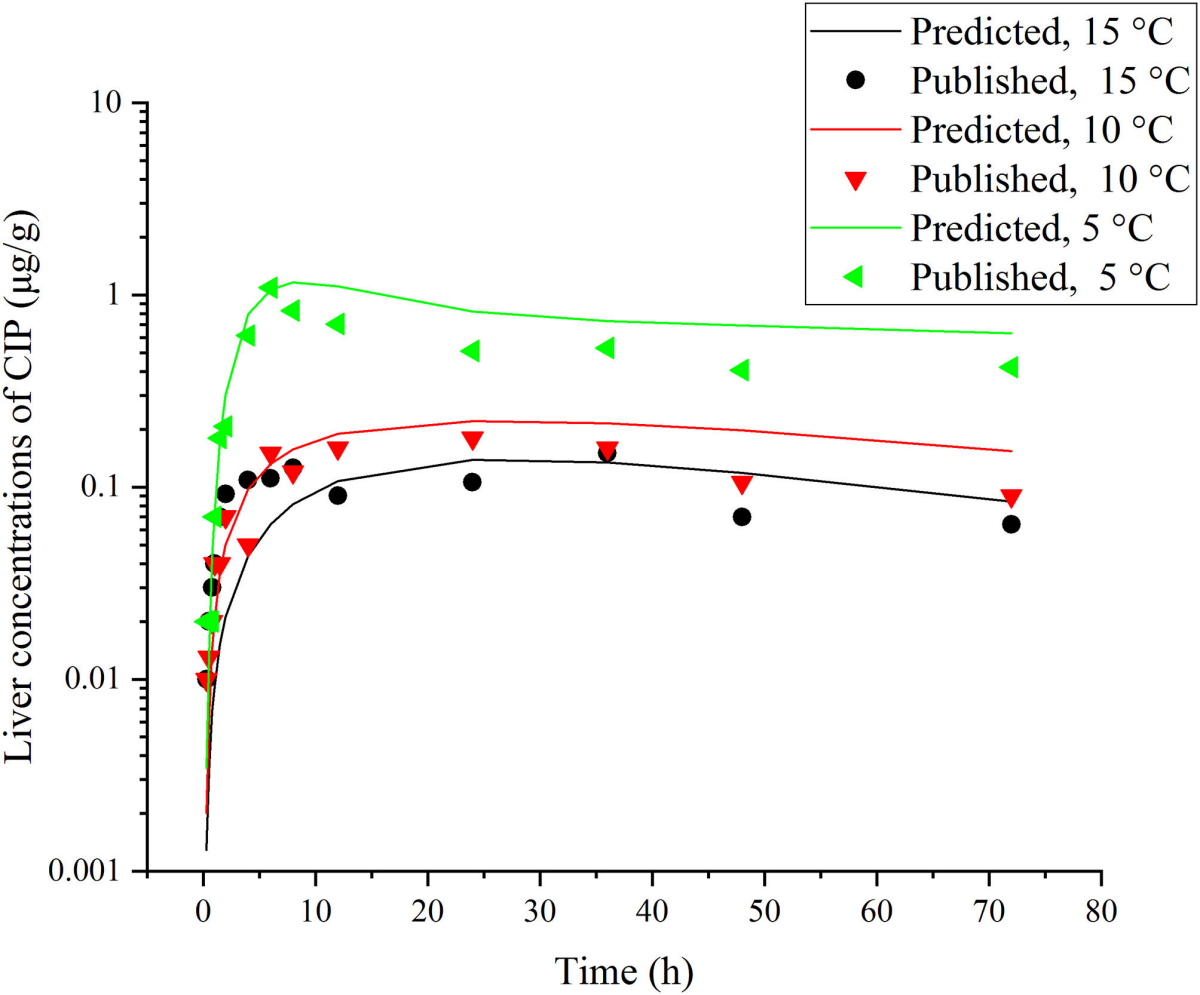
Comparisons between the predicted (curves) and published [points; (39)] CIP concentrations (μg/g) in the liver after a single oral dose of ENR at 30 mg/kg b.w. in rainbow trout reared at three different water temperature.

**Figure 6:**
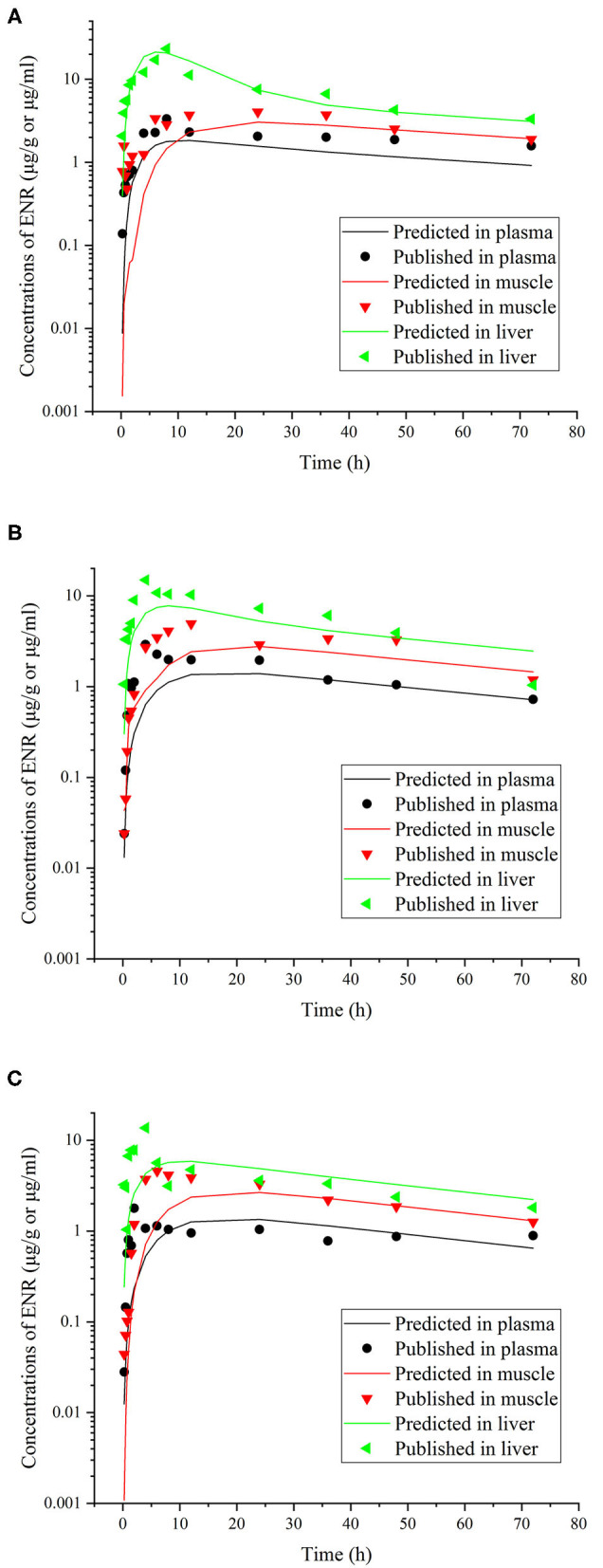
Comparisons between the predicted (curves) and published [points; (39)] ENR concentrations (μg/ml or μg/g) in plasma and tissues after a single oral dose of ENR at 30 mg/kg b.w. in rainbow trout reared at two different water temperatures [**(A)**, at 5°C; **(B)**, at 10°C; **(C)**, at 15°C].

**Figure 7:**
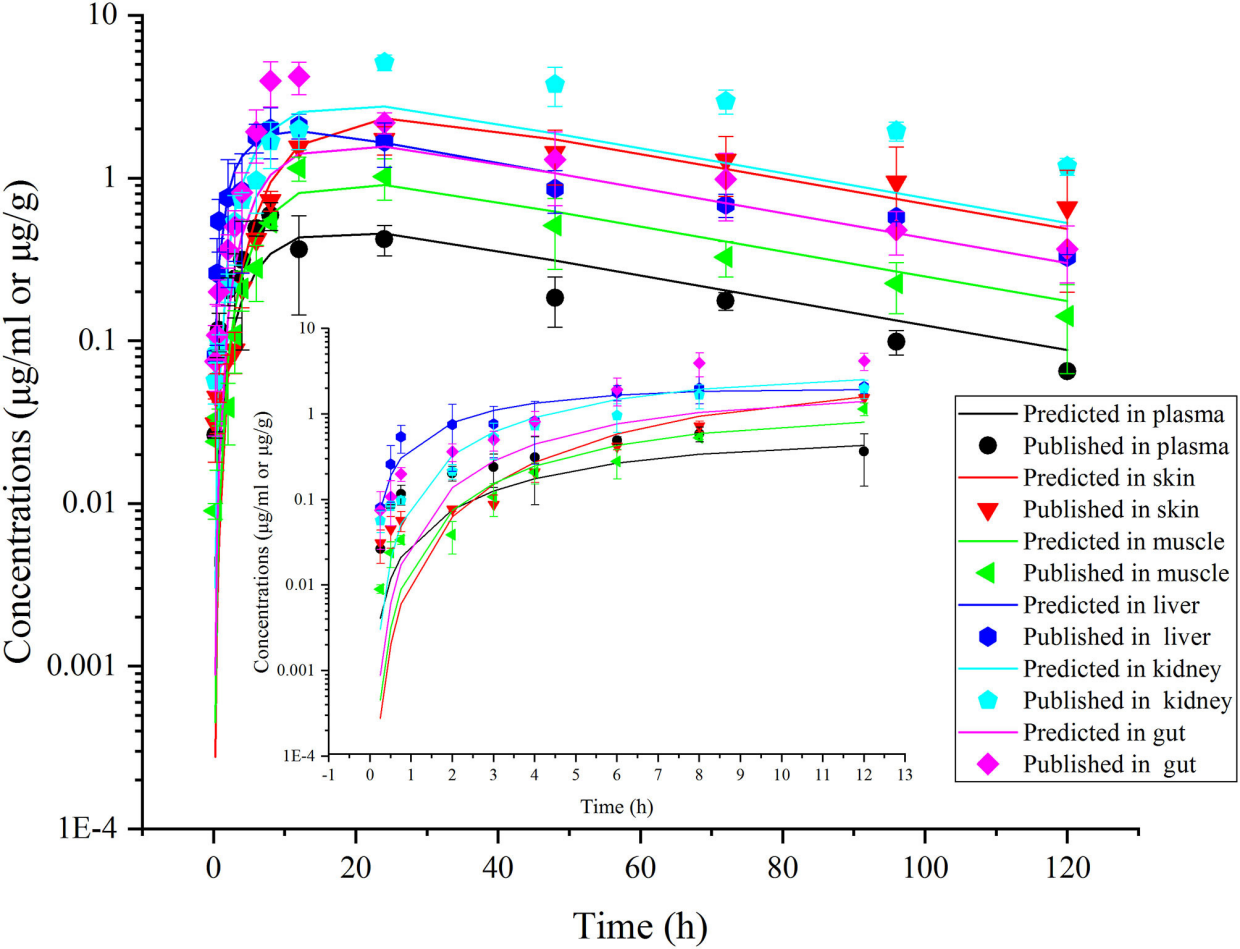
Comparisons between the predicted (curves) and published [points; (38)] ENR concentrations (μg/ml or μg/g) in plasma and tissues after a single oral dose of ENR at 10 mg/kg b.w. in rainbow trout reared at 16.3°C.

This model was validated through the visual comparison between the predicted and published concentrations of ENR in tissues/plasma or CIP in the liver, and the comparison results are shown in Figures 2–7. As demonstrated by these figures, those published ENR and CIP concentrations in plasma, serum, and tissues were all well-predicted after three different routes of administration and at different water temperatures. At the same time points, most predictions are very close to the reported concentrations (Figures 2–7). However, the CIP concentrations in the liver were overestimated by ~1.5 times, especially at the later sampling time points (Figure 5). This inconsistency might be because there were not enough published CIP concentration data for parameter optimization (Table 1). In the previous studies (3, 9, 13), the concentrations of CIP were usually irregular after different routes administration of ENR. Besides visual comparisons, the linear regression analysis was also undertaken between the predicted and published concentrations (Table 5). The current model was proved to be generally acceptable since most of the determination coefficients were above 0.75 except for some individual ones lower than (but very close to) 0.75. The smallest coefficient of determination (0.035) was determined for ENR concentrations in the liver (Table 5). The MAPE values were also calculated to evaluate the current model, and the results were similar to visual comparisons and linear regression analysis. The MAPE values ranged from 3.2 to 61%, and only four values >50% (Table 5). All these validation results confirmed that the present prediction results were acceptable.

**Table 5:** Results of linear regression analysis between the predictions and observations of ENR or CIP (μg/g or μg/ml) in tissues, plasma, and serum after different routes of administration in rainbow trout.

**Route of administration**	**Ref**.	**Water temperature –dose^**a**^**	**Matrix**	**Compound**	**Slope (a)**	**Intercept (b)**	***R*^**2**^**	**MAPE (%)**
IV	(3)	10–10	Plasma	ENR	1.004	0.006	0.997	3.20
PO	(37)	15–5	Serum	ENR	1.003	−0.0001	0.962	9.04
		15–10	Serum	ENR	1.223	−0.194	0.820	40.12
		15–50	Serum	ENR	0.997	0.039	0.949	7.97
		10–5	Serum	ENR	1.11	−0.07	0.878	17.03
		10–10	Serum	ENR	1.129	−0.104	0.802	23.32
		10–50	Serum	ENR	0.994	0.019	0.935	6.28
	(38)	16.3–10	Plasma	ENR	0.872	0.075	0.591	48.24
		16.3–10	Skin	ENR	0.815	0.076	0.933	42.29
		16.3–10	Muscle	ENR	1.144	−0.055	0.906	43.16
		16.3–10	Liver	ENR	0.972	0.011	0.902	18.92
		16.3–10	Kidney	ENR	1.346	0.071	0.651	48.7
		16.3–10	Gut	ENR	2.179	−0.03	0.692	52.26
IB	(38)	16.3–20	Plasma	ENR	1.077	0.007	0.524	18.17
		16.3–20	Skin	ENR	0.802	0.102	0.853	32.49
		16.3–20	Muscle	ENR	0.879	0.027	0.523	31.68
		16.3–20	Liver	ENR	0.503	0.156	0.566	32.39
		16.3–20	Kidney	ENR	0.761	−0.0002	0.884	39.33
		16.3–20	Gut	ENR	1.186	0.177	0.708	41.51
PO	(39)	15–30	Liver	CIP	0.570	0.043	0.491	55.64
		10–30	Liver	CIP	0.662	0.015	0.852	44.38
		5–30	Liver	CIP	0.740	−0.001	0.909	45.37
		15–30	Plasma	ENR	0.343	0.639	0.156	49.91
		15–30	Muscle	ENR	1.211	0.688	0.510	54.48
		15–30	Liver	ENR	0.321	3.858	0.035	49.43
		10–30	Plasma	ENR	1.061	0.583	0.420	48.14
		10–30	Muscle	ENR	1.520	0.196	0.723	35.80
		10–30	Liver	ENR	1.431	0.728	0.765	49.64
		5–30	Plasma	ENR	1.332	0.285	0.912	49.26
		5–30	Muscle	ENR	0.936	1.031	0.759	61.00
		5–30	Liver	ENR	0.725	2.245	0.852	30.21

a *This column was used to distinguish the concentrations data at different water temperatures or varying doses. The unites of water temperature and dose were °C and mg/kg BW (ppm in water). More details could be found in Table 1*.

### Sensitivity Analysis

The complete results of sensitivity analysis are presented in Supplementary Tables 2, 3. A positive NSC value indicated a positive correlation between the parameter value and the AUC, while a negative NSC indicated an opposite relationship between them. It was shown that most of the parameters have similar impact extents on ENR or CIP AUCs in different tissues. However, the parameters of Q_ck_, Q_cs_, V_cs_, P_l_, P_k_, P_s_, and P_g_ only had positive impacts on the AUCs of ENR (Supplementary Table 2). Similar positive influences on the CIP AUCs were observed for P_ml_ and P_mk_ (Supplementary Table 3). The dosage of different routes and the volume of aquaculture water had the highest influences on both AUCs of ENR and CIP (Supplementary Tables 2, 3) because these parameters directly represented the exposure doses of ENR. In addition to these exposure parameters, water temperature also had a more significant impact on the AUCs of ENR and CIP. This result was consistent with the blood flow limited mechanism because the cardiac output is proportional to water temperature (42).

### Monte Carlo Analysis and Withdrawal Interval Estimation

Monte Carlo analysis was only performed on the influential parameters (Supplementary Table 1). ENR and CIP concentrations and their total residues after one single PO administration of ENR at 10 mg/kg b.w. were predicted according to each Monte Carlo run. Results are shown in Supplementary Figures 1–9. Oral multiple-dose were also simulated. However, only the predicted concentrations at 16°C are shown in Supplementary Figures 10–12. In addition to PO administration, two immersion baths were also simulated at three different water temperatures; however, only the results at 16°C are shown in Supplementary Figures 13–18. Based on these predicted results, the withdrawal intervals were determined to ensure that the sum of ENR and CIP concentrations in the 99th percentile of the population (at least 495 virtual individuals) were below the corresponding MRLs.

**Figure 8:**
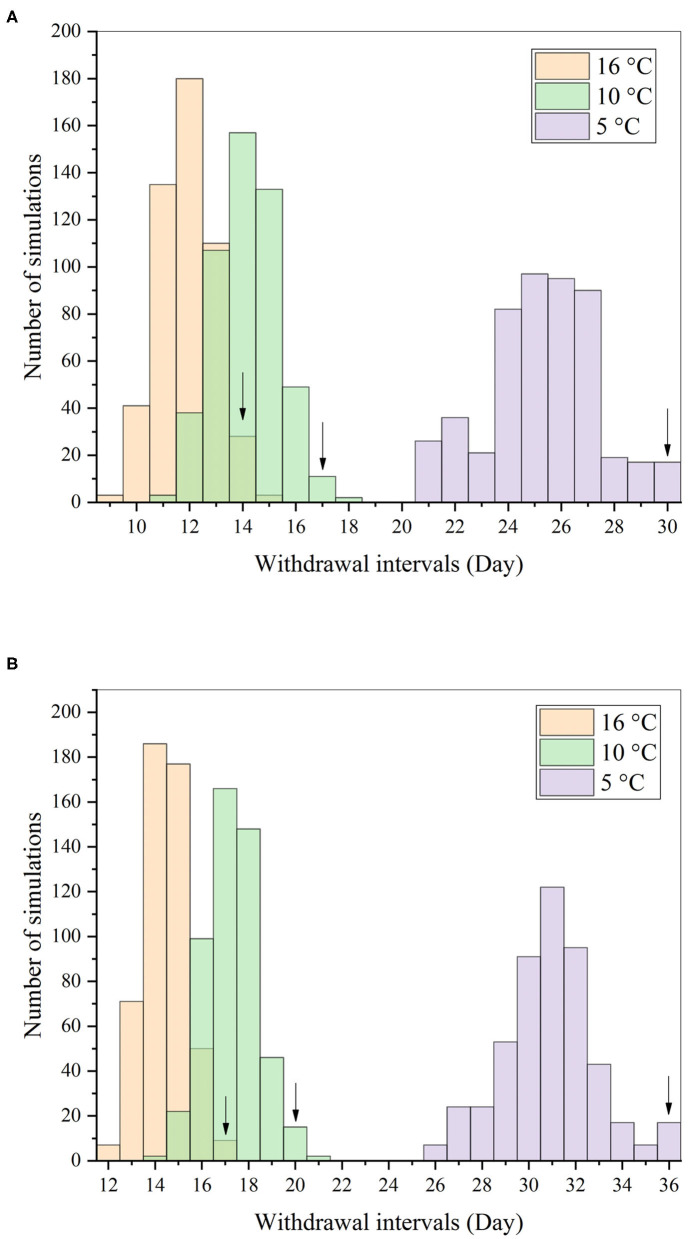
Distributions of withdrawal intervals in the muscle **(A)** and skin **(B)** after repeated oral dosing of ENR (20 mg/kg b.w. per day for 7 consecutive days) based on the Monte Carlo analysis. Notes: the arrows represented the 99th percentiles of the distribution at three different water temperatures.

The distributions of withdrawal intervals at different water temperatures were compared, and the results after multiple oral doses are shown in Figure 8 as examples. Those under the other dosage regimens could be found in the Supplementary Materials (Supplementary Figures 19, 20). Also, the comparison result of withdrawal intervals under different dosage regimens and water temperatures are presented in Table 6. Under the same conditions, the withdrawal interval in the skin was indeed longer than that in the muscle. The maximum difference between the two was 6 days, and the minimum was 2 days (Table 6). The present findings were consistent with the previous report (44). We also translated the unit of withdrawal interval under different dosage regimens from days to degree-days by multiplying the drug withdrawal interval in days by the corresponding water temperature. Their corresponding results were all shorter than the official withdrawal period in China (500 degree-days).

**Table 6:** Comparison of the withdrawal intervals under different dosage regimens calculated based on the current PBPK model.

**Dosage regimens**	**Water temperature (°C)**	**Muscle**		**Skin**	
		**Days**	**Degree-days**	**Days**	**Degree-days**
Single oral dose (10 mg/kg BW)	5	18	90	24	120
	10	11	110	14	140
	16	9	144	12	192
Multiple oral dose (20 mg/kg BW per day for 7 consecutive days)	5	30	150	36	180
	10	17	170	20	200
	16	14	224	17	272
An immersion bath in water with ENR concentration at 20 ppm for 2.5 h	5	16	80	22	110
	10	9	90	12	120
	16	8	128	10	160
An immersion bath in water with ENR concentration at 100 ppm for 0.5 h	5	26	130	31	155
	10	15	150	18	180
	16	13	208	15	240

## Discussion

Compared with those previous PBPK models in fish species (24, 25, 33), the present is the first one related to the water temperature. Unlike mammals, fish are heterothermic. The water temperature has a significant effect on the elimination of drugs from fish (19). Therefore, a PBPK model related to the water temperature will be of great significance. The validation results indicated that the present model could accurately predict the ENR and CIP concentrations in the water temperature range of 5°C to16°C. In the current model, three administration routes were simulated, containing PO, IB, and IV dosing. The first two routes were labeled by the regulatory authorities. The third one, although unapproved, was also simulated to optimize and validate the current model. In order to simulate the IB route, the aquaculture water was incorporated into the present model. Following the IB exposure, ENR was absorbed into the fish body from the water; after the distribution and metabolism, they were eventually excreted into the water underwent final degradation there. As the marker residue for ENR, parent compound and CIP were both simulated, and they were linked through the hepatic metabolism. The parameter of K_f_ was used to simulate the hepatic metabolism of ENR, and its final value (0.0725 h^−1^) was obtained by optimization. In the optimization process, the setting of the initial value is a key step. We initially conducted an extensive literature search to obtain the initial value of this parameter in fish. Unfortunately, it was not available. Therefore, we finally set it as 0.02 h^−1^. Some further studies, such as *in vitro* or *in vivo* metabolism experiments, should be carried out to validate these initial and final values for K_f_.

Monte Carlo simulation containing 500 iterations was included in this model to generate a population of 500 virtual individuals and to predict the drug concentrations in the population further. Based on these predicted concentrations, the withdrawal intervals were estimated and compared for different water temperatures. Compared with the conventional animal experiment method to calculate the drug withdrawal period, the PBPK model incorporating Monte Carlo simulation has three significant advantages. First, the PBPK model is predictive rather than descriptive, and its results are more accurate. Second, Monte Carlo simulation is not limited by the number of animals, and can fully take into account the variation between individual animals. Finally, the PBPK model can obtain a withdrawal interval closer to the clinical reality by changing the physiological parameters in the pathological state.

In the current model, most anatomical and physiological parameters in rainbow trout were from some previous fish models (24, 25, 33). It was found that all these parameters were valid because the present model had good predictions in most tissues after three routes of dosing. In addition to these parameters, the partition coefficients for ENR were determined by the area method (43). Those for CIP were assumed to be equal to the humans model (40). The final values of ENR partition coefficients ranged from 2.83 in muscle to 11.53 in the kidney (Table 3), and the corresponding values for CIP ranged from 0.718 in the skin to 8.2 in the kidney (Table 3). These results indicated that the kidney was a primary elimination organ for both ENR and CIP, consistent with the previous study (23). Parameters values regarding to extravascular absorption were acquired through parameter optimization. And the values of K_st_, K_aPO_, and K_aIB_ were 0.175, 0.052, and 1.103, respectively (Table 4). These results showed that ENR had a relatively quicker absorption following an immersion bath, which might be related to the structure of the current model. Following IB exposure, the ENR was directly absorbed into gills from the aquaculture water, while after PO dosing, ENR firstly entered the stomach and could only be absorbed after entering the intestine under the action of gastric emptying. The parameters interrelated to the elimination of ENR were also obtained through optimization, and the values of K_f_, Cl_re_, K_guc_, and K_gw_ were 0.0725 h^−1^, 0.058 L/kg/h, 0.605 h^−1^, and 0.061 h^−1^, respectively (Table 4). These parameter values indicated that the renal and intestinal excretions for ENR were more critical than the biotransformation, which was also consistent with the fact that the CIP concentrations were much lower than those of ENR after different routes administration of ENR (3, 37, 38). CIP had a quicker elimination than ENR with a Cl_mre_ of 116.14 L/h/kg.

Water temperature has a significant effect on the drug disposition in the fish body (19), for two main reasons. First, water temperature significantly affects the cardiac output, distribution of blood flow, and local perfusion in different tissues (42). Like ENR, the distributions of most drugs are restricted by the blood flow rate. Second, as heterothermic animals, fish cannot control their body temperature. It is well-known that lower temperatures can reduce the activity of metabolic enzymes, thereby slowing down the rate of metabolic reactions. Therefore, the water temperature will affect the distribution and metabolism of drugs in fish. In this model, we use the relationship between water temperature and cardiac output previously reported by other researchers (42) to estimate the temperature effects on drug disposition. We have also tried to incorporate the impact of water temperature on ENR metabolism in the model. However, a previous study showed that water temperature had little effect on the ENR tissues concentrations in rainbow trout, and only slightly affected ENR concentration in plasma (39). Also, the expression amount of CYP3A in rainbow trout liver cells did not differ significantly at three different temperatures (5, 10, and 15°C) (39). Therefore, in the current model, the water temperature was only linked to cardiac output (Table 2).

There are several limitations to the current study. The first one is related to the optimization of parameters related to drug absorption and elimination. Because of the abundance of ENR and CIP concentration data after different routes of administration in rainbow trout, we did not carry out relevant experiments to obtain these parameter values but obtained them through parameter optimization based on those published concentration data. Whilst this is not necessarily incorrect, more studies should be further performed to validate these parameter values. The second limitation pertains to the aquaculture water compartment. Although the culture water compartment was included in the current model, neither ENR nor CIP concentrations were predicted or validated in the water. This is mainly because the volume of aquaculture water was not available in the previous studies (2, 3, 37, 39), so it is impossible to predict the drug concentration in the water. Also, this study aimed to predict drug concentrations in edible tissues to reduce residual risk of ENR and CIP. Therefore, the water concentrations were not expected here. Thirdly, the predicted concentrations, such as ENR and CIP concentrations in the gill, CIP concentration in tissues other than the liver, were not validated because of the scarcity of observations. In the present model, the gill is a bridge connecting venous and arterial blood. More importantly, gill is also the main route of drug absorption after IB exposure. In order to improve this limitation, some depletion studies of ENR following different routes of administration and at different water temperatures should be further performed to obtain the ENR and CIP concentrations in different tissues to validate the current model. Fourthly, the age of rainbow trout was not taken into account, which might affect the metabolism of ENR. The withdrawal time of enrofloxacin at different water temperatures for different administration routes varied from 8 to 36 days (Table 6). While the maximum recorded lifespan for a rainbow trout was up to 11 years. Therefore, we have reason to believe that a withdrawal period of 38 days may not have any significant effect on ENR metabolism during the lifespan. Finally, the partition coefficients for CIP were assumed to be the same as those for humans. This is a major assumption that could be erroneous due to differences in the phospholipid and protein composition of tissues between humans and fish. However, because the concentration data of CIP in rainbow trout tissue were relatively scarce, we used those available concentration data to optimize and validate the model, rather than to calculate the partition coefficients for CIP. Some depletion studies should be further performed to validate these partition coefficients for CIP.

The withdrawal intervals were estimated and compared at different water temperatures based on this model (Table 5). Under different dosage regimens, all withdrawal intervals at low water temperatures were longer than those at high water temperatures. This was an obvious fact, and its reason stemmed from the influence mentioned above of water temperature on the cardiac output. It should be noted that the withdrawal intervals estimated here, ranging from 80 to 272 degree-days (Table 5), were all shorter than the labeled one in China (500 degree-days). The officially approved target tissue for ENR residue was muscle and skin in natural proportions in fish species. However, the skin and muscle were separated in the present model because a previous study showed that ENR seemed to be especially bound to fish skin (44). Our predicted results indeed proved this specific binding (Table 5). Therefore, the present results might encourage the regulatory authorities to shorten the official withdrawal period for ENR in rainbow trout.

## Conclusion

In conclusion, the current model can adequately predict ENR and CIP concentrations following three routes of dosing in rainbow trout reared at a wide range of water temperatures (5–16°C). The Monte Carlo analysis was included in the model to predict tissue depletion in a population containing 500 virtual individuals and to further determine the withdrawal intervals for ENR in rainbow trout at three different water temperatures. This research provides a basis for application the temperature-related PBPK model to predict residue concentrations and further estimate the withdrawal intervals at different water temperatures. This model also can be further used for a risk assessment for some water pollutants.

## Data Availability Statement

The original contributions presented in the study are included in the article/Supplementary Materials, further inquiries can be directed to the corresponding author/s.

## Ethics Statement

Ethical review and approval was not required for the animal study because this was a modeling study, and no animal experiments were involved in this study.

## Author Contributions

FanY conceived this project. FangY and C-SZ did the citation research. DW, HW, and Z-WS extracted and collected all the concentration-time data. H-TS and MZ collected the physiological and anatomical parameters for rainbow trout. FanY developed and optimized this model with support from M-LY and YZ. FanY wrote the manuscript with the backing from FangY, C-SZ, and HW. All authors read and approved this final manuscript.

## Conflict of Interest

The authors declare that the research was conducted in the absence of any commercial or financial relationships that could be construed as a potential conflict of interest.

## References

[1] FAO The State of World Fisheries and Aquaculture 2020. (2020). Available online at: www.fao.org/3/ca9229en/ca9229en.pdf (accessed September 10, 2020).

[2] Lucchetti D Fabrizi L Guandalini E Podesta E Marvasi L Zaghini A. Long depletion time of enrofloxacin in rainbow trout (*Oncorhynchus mykiss*). Antimicrob Agents Chemother. (2004) 48:3912–7. 10.1128/AAC.48.10.3912-3917.2004 15388452 PMC521881

[3] Liang J Li J Zhao F Liu P Chang Z Pharmacokinetics and tissue behavior of enrofloxacin and its metabolite ciprofloxacin in turbot *Scophthalmus maximus* at two water temperatures. Chin J Oceanol Limnol. (2012) 30:644–53. 10.1007/s00343-012-1228-2

[4] Fauzi IA Haga Y Kondo H Hirono I Satoh S Dietary citrulline improves survival of rainbow trout *Oncorhynchus mykiss* juveniles challenged with *Vibrio anguillarum*. Aquaculture. (2020) 528:735491 10.1016/j.aquaculture.2020.735491

[5] Kiadaliri M Firouzbakhsh F Deldar H Effects of feeding with red algae (*Laurencia caspica*) hydroalcoholic extract on antioxidant defense, immune responses, and immune gene expression of kidney in rainbow trout (*Oncorhynchus mykiss*) infected with *Aeromonas hydrophila*. Aquaculture. (2020) 526:735361 10.1016/j.aquaculture.2020.735361

[6] Bulfon C Prearo M Volpatti D Byadgi O Righetti M Maniaci MG Resistant and susceptible rainbow trout (*Oncorhynchus mykiss*) lines show distinctive immune response to *Lactococcus garvieae*. Fish Shellfish Immunol. (2020) 105:457–68. 10.1016/j.fsi.2020.06.040 32673645

[7] Samuelsen OB Pharmacokinetics of quinolones in fish: a review. Aquaculture. (2006) 255:55–75. 10.1016/j.aquaculture.2005.12.008

[8] Quesada SP Paschoal JAR Reyes FGR. Considerations on the aquaculture development and on the use of veterinary drugs: special issue for fluoroquinolones-a Review. J Food Sci. (2013) 78:R1321-R1333. 10.1111/1750-3841.12222 23909512

[9] Yang F Kang J Yang F Zhao Z Kong T Zeng Z. Preparation and evaluation of enrofloxacin microspheres and tissue distribution in rats. J Vet Sci. (2015) 16:157–64. 10.4142/jvs.2015.16.2.157 25643802 PMC4483498

[10] Darwish AM Farmer BD Hawke JP. Improved method for determining antibiotic susceptibility of *Flavobacterium columnare* isolates by broth microdilution. J Aquat Anim Health. (2008) 20:185–91. 10.1577/H07-047.1 19306607

[11] Hsu H-M Bowser PR Schachte Jr JH, Scarlett JM, Babish JG. Winter field trials of enrofloxacin for the control of *Aeromonas salmonicida* infection in salmonids. J World Aquac Soc. (1995) 26:307–14. 10.1111/j.1749-7345.1995.tb00259.x

[12] Hsu H-M Wooster GA Bowser PR Efficacy of enrofloxacin for the treatment of salmonids with bacterial kidney disease, caused by *Renibacterium salmoninarum*. J Aquat Anim Health. (1994) 6:220–3. 10.1577/1548-8667(1994)006<0220:EOEFTT>2.3.CO;2

[13] Kyuchukova R Milanova A Pavlov A Lashev L. Comparison of plasma and tissue disposition of enrofloxacin in rainbow trout (*Oncorhynchus mykiss*) and common carp (*Cyprinus carpio*) after a single oral administration. Food Addit Contam Part A Chem Anal Control Expo Risk Assess. (2015) 32:35–9. 10.1080/19440049.2014.983998 25372241

[14] Koc F Uney K Atamanalp M Tumer I Kaban G Pharmacokinetic disposition of enrofloxacin in brown trout (*Salmo trutta fario*) after oral and intravenous administrations. Aquaculture. (2009) 295:142–4. 10.1016/j.aquaculture.2009.06.004

[15] Commission of Chinese Veterinary Pharmacopoeia Enrofloxacin powder (for aquaculture application). (2020). Available online at: http://124.126.15.169:8081/cx/# (accessed September 10, 2020).

[16] Okocha RC Olatoye IO Adedeji OB. Food safety impacts of antimicrobial use and their residues in aquaculture. Public Health Reviews. (2018) 39:21. 10.1186/s40985-018-0099-2 30094087 PMC6081861

[17] Mo WY Chen Z Leung HM Leung AOW. Application of veterinary antibiotics in China's aquaculture industry and their potential human health risks. Environ Sci Pollut Res Int. (2017) 24:8978–89. 10.1007/s11356-015-5607-z 26498964

[18] European Union (EU). Directive 2004/28/EC of the European Parliament and of the Council of 31 March 2004 amending Directive 2001/82/EC on the Community code relating to veterinary medicinal products (Text with EEA relevance). (2004). Available online at: https://eur-lex.europa.eu/legal-content/EN/TXT/?uri=celex%3A32004L0028 (accessed September 10, 2020).

[19] Yang F Yang F Wang G Kong T Wang H Zhang C Effects of water temperature on tissue depletion of florfenicol and its metabolite florfenicol amine in crucian carp (*Carassius auratus gibelio*) following multiple oral doses. Aquaculture. (2020) 515:734542 10.1016/j.aquaculture.2019.734542

[20] Barani A Fallah AA HPLC analysis of some allowable-antibiotic multiresidues in farmed rainbow trout in Iran. Toxin Rev. (2015) 34:206–9. 10.3109/15569543.2015.1116097

[21] Yang F Huang XH Li GH Ni HJ Zhao YD Ding HZ. Estimating tulathromycin withdrawal time in pigs using a physiologically based pharmacokinetics model. Food Addit Contam Part A Chem Anal Control Expo Risk Assess. (2013) 30:1255–63. 10.1080/19440049.2013.797113 23767965

[22] Yang F Si HB Wang YQ Zhao ZS Zhou BH Hao XQ. Pharmacokinetics of doxycycline in laying hens after intravenous and oral administration. Br Poult Sci. (2016) 57:576–80. 10.1080/00071668.2016.1184228 27137900

[23] European Medicines Agency (EMA). Enrofloxacin: Summary report (1)—Committee for Veterinary Medicinal Products. (1996). Available onlineat: https://www.ema.europa.eu/en/documents/mrl-report/enrofloxacin-summary-report-1-committee-veterinary-medicinal-products_en.pdf (accessed September 10, 2020).

[24] Yang F Sun N Sun YX Shan Q Zhao HY Zeng DP. A physiologically based pharmacokinetics model for florfenicol in crucian carp and oral-to-intramuscular extrapolation. J Vet Pharmacol Ther. (2013) 36:192–200. 10.1111/j.1365-2885.2012.01419.x 22712485

[25] Xu N Li M Chou WC Lin Z. A physiologically based pharmacokinetic model of doxycycline for predicting tissue residues and withdrawal intervals in grass carp (*Ctenopharyngodon idella*). Food Chem Toxicol. (2020) 137:111127. 10.1016/j.fct.2020.111127 31945393

[26] Yang F Yang F Shi W Si H Kong T Wang G. Development of a multiroute physiologically based pharmacokinetic model for orbifloxacin in rabbits. J Vet Pharmacol Ther. (2018) 41:622–31. 10.1111/jvp.12496 29457247

[27] Leavens TL Tell LA Kissell LW Smith GW Smith DJ Wagner SA. Development of a physiologically based pharmacokinetic model for flunixin in cattle (*Bos taurus*). Food Addit Contam Part A Chem Anal Control Expo Risk Assess. (2014) 31:1506–21. 10.1080/19440049.2014.938363 25082521

[28] Yang F Sun N Liu YM Zeng ZL. Estimating danofloxacin withdrawal time in broiler chickens based on physiologically based pharmacokinetics modeling. J Vet Pharmacol Ther. (2015) 38:174–82. 10.1111/jvp.12162 25236844

[29] Yang F Yang YR Wang L Huang XH Qiao G Zeng ZL. Estimating marbofloxacin withdrawal time in broiler chickens using a population physiologically based pharmacokinetics model. J Vet Pharmacol Ther. (2014) 37:579–88. 10.1111/jvp.12137 24903645

[30] Li M Gehring R Riviere JE Lin Z. Development and application of a population physiologically based pharmacokinetic model for penicillin G in swine and cattle for food safety assessment. Food Chem Toxicol. (2017) 107:74–87. 10.1016/j.fct.2017.06.023 28627373

[31] Li M Gehring R Riviere JE Lin Z Probabilistic physiologically based pharmacokinetic model for penicillin G in milk from dairy cows following intramammary or intramuscular administrations. Toxicol Sci. (2018) 164:85–100. 10.1093/toxsci/kfy067 29945226

[32] Lin Z Gehring R Mochel JP Lave T Riviere JE. Mathematical modeling and simulation in animal health—Part II: principles, methods, applications, and value of physiologically based pharmacokinetic modeling in veterinary medicine and food safety assessment. J Vet Pharmacol Ther. (2016) 39:421–38. 10.1111/jvp.12311 27086878

[33] Abbas R Hayton WL. A physiologically based pharmacokinetic and pharmacodynamic model for paraoxon in rainbow trout. Toxicol Appl Pharmacol. (1997) 145:192–201. 10.1006/taap.1997.8168 9221837

[34] Erickson RJ McKim JM A simple flow-limited model for exchange of organic chemicals at fish gills. Environ Toxicol Chem. (1990) 9:159–65. 10.1002/etc.5620090205

[35] Nichols JW McKim JM Andersen ME Gargas ML Clewell HJ Erickson RJ. A physiologically based toxicokinetic model for the uptake and disposition of waterborne organic chemicals in fish. Toxicol Appl Pharmacol. (1990) 106:433–47. 10.1016/0041-008x(90)90338-u 2260091

[36] Salmina ES Wondrousch D Kuhne R Potemkin VA Schuurmann G. Variation in predicted internal concentrations in relation to PBPK model complexity for rainbow trout. Sci Total Environ. (2016) 550:586–97. 10.1016/j.scitotenv.2016.01.107 26849323

[37] Bowser PR Wooster GA St Leger J Babish JG. Pharmacokinetics of enrofloxacin in fingerling rainbow trout (*Oncorhynchus mykiss*). J Vet Pharmacol Ther. (1992) 15:62–71. 10.1111/j.1365-2885.1992.tb00987.x 1315398

[38] Urzua N Messina MJ Prieto G Luders C Errecalde C. Pharmacokinetics and tissue disposition of enrofloxacin in rainbow trout after different routes of administration. Xenobiotica. (2020) 50:1236–41. 10.1080/00498254.2020.1747119 32208796

[39] Pan H Effect of water velocity, temperature and light intensity on pharmacokinetics of enrofloxacin in rainbow (dissertation/master's thesis). Shanghai Ocean University, Shanghai (2017).

[40] Sadiq MW Nielsen EI Khachman D Conil JM Georges B Houin G. A whole-body physiologically based pharmacokinetic (WB-PBPK) model of ciprofloxacin: a step towards predicting bacterial killing at sites of infection. J Pharmacokinet Pharmacodyn. (2017) 44:69–79. 10.1007/s10928-016-9486-9 27578330 PMC5376394

[41] Yang F Lin Z Riviere JE Baynes RE. Development and application of a population physiologically based pharmacokinetic model for florfenicol and its metabolite florfenicol amine in cattle. Food Chem Toxicol. (2019) 126:285–94. 10.1016/j.fct.2019.02.029 30825586

[42] Barron MG Tarr BD Hayton WL Temperature-dependence of cardiac output and regional blood flow in rainbow trout, *Salmo gairdneri* Richardson. J Fish Biol. (1987) 31:735–44. 10.1111/j.1095-8649.1987.tb05276.x

[43] Gallo JM Lam FC Perrier DG. Area method for the estimation of partition coefficients for physiological pharmacokinetic models. J Pharmacokinet Biopharm. (1987) 15:271–80. 10.1007/BF01066322 3668804

[44] Steffenak I Hormazabal V Yndestad M. Reservoir of quinolone residues in fish. Food Addit Contam. (1991) 8:777–80. 10.1080/02652039109374035 1667390

