# Supplementary material for: Development and Application of a Water Temperature Related Physiologically Based Pharmacokinetic Model for Enrofloxacin and Its Metabolite Ciprofloxacin in Rainbow Trout
Source: Front Vet Sci. 2021 Jan 25;7:608348. doi: 10.3389/fvets.2020.608348 (PMC7874017; doi:10.3389/fvets.2020.608348)
Supplement: Supplementary file 1 [file Data_Sheet_1.docx]

Development and application of a water temperature related physiologically based pharmacokinetic model for enrofloxacin and its metabolite ciprofloxacin in rainbow trout

Fan Yang^1,2*^, Fang Yang^1^, Dan Wang^3^, Chao-Shuo Zhang^1^, Han Wang^1^, Zhe-Wen Song^1^, Hao-Tian Shao^1^, Mei Zhang^1^, Meng-Li Yu^1^, Yang Zheng^1^

^1^ College of Animal Science and Technology, Henan University of Science and Technology, Luoyang 471023, China

^2^ Environmental and Animal Products Safety Laboratory of Key Discipline in University of Henan Province, Henan University of Science and Technology, Luoyang 471023, China

^3^ Jiaozuo Livestock Product Quality and Safety Monitoring Center, Jiaozuo 454003, China

**Supplementary Materials**

[**1 PBPK model code written in acslXtreme software** 3](#_Toc51474077)

[**2 M-type code file used to calculate the withdrawal intervals** 9](#_Toc51474078)

[**3 Supplementary Tables** 10](#_Toc51474079)

[**Table S1.** The distribution information of those influential parameters subjected to Monte Carlo analysis 10](#_Toc51474080)

[**Table S2.** Normalized sensitivity coefficients of all 48 parameters for the sensitivity analysis about the influence of model parameters on the AUCs of enrofloxacin (ENR) 11](#_Toc51474081)

[**Table S3.** Normalized sensitivity coefficients of all 48 parameters for the sensitivity analysis about the influence of model parameters on the AUCs of ciprofloxacin (CIP) 12](#_Toc51474082)

[**4 Supplementary Figures** 14](#_Toc51474083)

[**Figure S1**. Predicted ENR concentrations (μg/ml or μg/g) after a single oral dose of ENR at 10 mg/kg BW in rainbow trout reared at 5 °C based on the Monte Carlo analysis with 500 iterations: a plasma, b serum, c liver, d kidney, e muscle, f skin 14](#_Toc51474084)

[**Figure S2**. Predicted CIP concentrations (μg/ml or μg/g) after a single oral dose of ENR at 10 mg/kg BW in rainbow trout reared at 5 °C based on the Monte Carlo analysis with 500 iterations: a plasma, b serum, c liver, d muscle, e muscle, f skin 15](#_Toc51474085)

[**Figure S3**. Predicted total concentrations (μg/ml or μg/g) of ENR and CIP after a single oral dose of ENR at 10 mg/kg BW in rainbow trout reared at 5 °C based on the Monte Carlo analysis with 500 iterations: a plasma, b serum, c liver, d kidney, e muscle, f skin 16](#_Toc51474086)

[Note: The horizontal red straight line represents the MRL of ENR in the muscle (0.1 μg/g). 16](#_Toc51474087)

[**Figure S4**. Predicted ENR concentrations (μg/ml or μg/g) after a single oral dose of ENR at 10 mg/kg BW in rainbow trout reared at 10 °C based on the Monte Carlo analysis with 500 iterations: a plasma, b serum, c liver, d kidney, e muscle, f skin 16](#_Toc51474088)

[**Figure S5**. Predicted CIP concentrations (μg/ml or μg/g) after a single oral dose of ENR at 10 mg/kg BW in rainbow trout reared at 10 °C based on the Monte Carlo analysis with 500 iterations: a plasma, b serum, c liver, d kidney, e muscle, f skin 17](#_Toc51474089)

[**Figure S6**. Predicted total concentrations (μg/ml or μg/g) of ENR and CIP after a single oral dose of ENR at 10 mg/kg BW in rainbow trout reared at 10 °C based on the Monte Carlo analysis with 500 iterations: a plasma, b serum, c liver, d kidney, e muscle, f skin 18](#_Toc51474090)

[**Figure S7**. Predicted ENR concentrations (μg/ml or μg/g) after a single oral dose of ENR at 10 mg/kg BW in rainbow trout reared at 16 °C based on the Monte Carlo analysis with 500 iterations: a plasma, b serum, c liver, d kidney, e muscle, f skin 19](#_Toc51474091)

[**Figure S8**. Predicted CIP concentrations (μg/ml or μg/g) after a single oral dose of ENR at 10 mg/kg BW in rainbow trout reared at 16 °C based on the Monte Carlo analysis with 500 iterations: a plasma, b serum, c liver, d kidney, e muscle, f skin 19](#_Toc51474092)

[**Figure S9**. Predicted total concentrations (μg/ml or μg/g) of ENR and CIP after a single oral dose of ENR at 10 mg/kg BW in rainbow trout reared at 16 °C based on the Monte Carlo analysis with 500 iterations: a plasma, b serum, c liver, d kidney, e muscle, f skin 20](#_Toc51474093)

[**Figure S10**. Predicted ENR concentrations (μg/ml or μg/g) after multiple oral doses of ENR at 20 mg/kg BW per day for 7 consecutive days in rainbow trout reared at 16 °C based on the Monte Carlo analysis with 500 iterations: a plasma, b serum, c liver, d kidney, e muscle, f skin 21](#_Toc51474094)

[**Figure S11**. Predicted CIP concentrations (μg/ml or μg/g) after multiple oral doses of ENR at 20 mg/kg BW per day for 7 consecutive days in rainbow trout reared at 16 °C based on the Monte Carlo analysis with 500 iterations: a plasma, b serum, c liver, d kidney, e muscle, f skin 21](#_Toc51474095)

[**Figure S12**. Predicted total concentrations (μg/ml or μg/g) of ENR and CIP after multiple oral doses of ENR at 20 mg/kg BW per day for 7 consecutive days in rainbow trout reared at 16 °C based on the Monte Carlo analysis with 500 iterations: a plasma, b serum, c liver, d kidney, e muscle, f skin 22](#_Toc51474096)

[**Figure S13**. Predicted ENR concentrations (μg/ml or μg/g) after an immersion bath in water with ENR concentration at 20 ppm for 2.5 hours for rainbow trout reared at 16 °C based on the Monte Carlo analysis with 500 iterations: a plasma, b serum, c liver, d kidney, e muscle, f skin 23](#_Toc51474097)

[**Figure S14**. Predicted CIP concentrations (μg/ml or μg/g) after an immersion bath in water with ENR concentration at 20 ppm for 2.5 hours for rainbow trout reared at 16 °C based on the Monte Carlo analysis with 500 iterations: a plasma, b serum, c liver, d kidney, e muscle, f skin 23](#_Toc51474098)

[**Figure S15**. Predicted total concentrations (μg/ml or μg/g) of ENR and CIP after an immersion bath in water with ENR concentration at 20 ppm for 2.5 hours for rainbow trout reared at 16 °C based on the Monte Carlo analysis with 500 iterations: a plasma, b serum, c liver, d kidney, e muscle, f skin 24](#_Toc51474099)

[**Figure S16**. Predicted ENR concentrations (μg/ml or μg/g) after an immersion bath in water with ENR concentration at 100 ppm for 0.5 hours for rainbow trout reared at 16 °C based on the Monte Carlo analysis with 500 iterations: a plasma, b serum, c liver, d kidney, e muscle, f skin 25](#_Toc51474100)

[**Figure S17**. Predicted CIP concentrations (μg/ml or μg/g) after an immersion bath in water with ENR concentration at 100 ppm for 0.5 hours for rainbow trout reared at 16 °C based on the Monte Carlo analysis with 500 iterations: a plasma, b serum, c liver, d kidney, e muscle, f skin 25](#_Toc51474101)

[**Figure S18**. Predicted total concentrations (μg/ml or μg/g) of ENR and CIP after an immersion bath in water with ENR concentration at 100 ppm for 0.5 hours for rainbow trout reared at 16 °C based on the Monte Carlo analysis with 500 iterations: a plasma, b serum, c liver, d kidney, e muscle, f skin 26](#_Toc51474102)

[**Figure S19**. Distributions of withdrawal intervals in the muscle (a) and skin (b) after an immersion bath in water with ENR concentration of 20 ppm for 2.5 h based on the Monte Carlo analysis. Notes: the solid arrows indicated the 99th percentiles of the distribution at different water temperatures. 26](#_Toc51474103)

[**Figure S20**. Distributions of withdrawal intervals in the muscle (a) and skin (b) after an immersion bath in water with ENR concentration of 10 ppm for 0.5 h based on the Monte Carlo analysis. Notes: the solid arrows indicated the 99th percentiles of the distribution at different water temperatures. 27](#_Toc51474104)

[**Supplementary References** 27](#_Toc51474105)

**1 PBPK model code written in acslXtreme software**

PROGRAM

! This PBPK model was developed by Dr. Fan Yang in the summer of 2020.

INITIAL

!parameters of Cardiac output

constant Temp=5 ! water temperature, °C

CO=3.95*Temp-12.9 ! Cardiac output, ml/min/kg; ([1](#_ENREF_1)) References, the same below

Qtot=CO/1000*60*bw ! translate the CO unit from ml/min/kg to L/h

!body weight

constant bw=0.05 ! kg, equal to L

!parameters of tissue weight (fraction of BW) and blood flow (fraction of CO)

constant Vcgi=0.039, Qcgi=1 ! gill, ([2](#_ENREF_2))

constant Vck=0.00841,Qck=0.056 ! kidney, ([3](#_ENREF_3))

constant Vcm=0.66,Qcm=0.6 ! muscle, ([3](#_ENREF_3))

constant Vcs=0.1, Qcs=0.053 ! skin, ([2](#_ENREF_2))

constant Vcg=0.0852, Qcg=0.1539 ! gut, ([2](#_ENREF_2))

constant Vcl=0.0126, Qcl=0.029 ! liver, ([3](#_ENREF_3))

constant Vcvb=0.059 ! Venous blood, ([4](#_ENREF_4))

constant Vcab=0.015 ! Arterial blood, ([4](#_ENREF_4))

Vcr=1-(Vcgi+Vck+Vcm+Vcs+Vcg+Vcl+Vcvb+Vcab) ! the rest body, fraction of BW

Qcr=1-(Qck+Qcm+Qcs+Qcl+Qcg) ! the rest body, fraction of CO

constant pcv=0.304 ! Hematocrit, ([1](#_ENREF_1))

constant pre=0.555 ! 1 minus serum ratio

! translate the units of parameter (V and Q) listed above from fraction to real value

! tissue weight

Vgi=bw*Vcgi Vk=bw*Vck Vm=bw*Vcm Vs=bw*Vcs Vg=bw*Vcg

Vl=bw*Vcl Vvb=bw*Vcvb Vab=bw*Vcab Vr=bw*Vcr Vmr=Vr+Vg

! change the weight (volume) of blood to that of plasma

Vvp=Vvb*(1-pcv) Vap=Vab*(1-pcv)

! change the weight (volume) of blood to that of serum

Vvs=Vvb*(1-pre) Vas=Vab*(1-pre)

! blood flow

Qk=Qck*Qtot Qm=Qtot*Qcm Qs=Qtot*Qcs

Qg=Qcg*Qtot Ql=Qtot*Qcl Qr=Qtot*Qcr

! parameters for enrofloxacin (ENR)

! partition coefficient

constant Pgi=3.46,Pl=4.9,Pk=11.53,Pm=2.83,Ps=7.38,Pg=4.88,Pr=0.13

! These partition coefficients were calculated according to the area method ([5](#_ENREF_5)) based on the enrofloxacin concentrations in plasma and tissues (including muscle, skin, gut, kidney, and liver) derived from a previous report ([6](#_ENREF_6)). Three dosage regimens were applied in that study, including one oral dosing and two immersion baths with different drug concentrations. Therefore, we calculated the partition coefficient for enrofloxacin based on the average AUC values.

! Pr and Pgi were obtained by optimization.

! parameters for ciprofloxacin

! partition coefficient

constant Pmgi=2.45,Pml=3.67,Pmk=8.2,Pmm=1.6,Pms=0.718,Pmg=3.39,Pmr=0.15

! Partition coefficients for ciprofloxacin in the tissues of muscle, skin, gut, liver, and kidney in rainbow trout were assumed to be equal to those previously reported in humans ([7](#_ENREF_7)). Those in the other compartments (gill and the rest of body) were optimized using a maximum likelihood algorithm of Nelder-Mead based on the concentrations of ciprofloxacin in serum, muscle, and liver, derived from a previous report ([8](#_ENREF_8)).

! oral absorpation

constant Kst=0.175, KaPO=0.052 ! optimization

! Kst (1/h) is the rate constant of gastric emptying, and KaPO (1/h) is the absorption rate constant.

constant F=0.6613 ! Oral bioavailability; ([9](#_ENREF_9))

! IB absorption

constant KaIB=1.103 ! 1/h

! elimination for enrofloxacin

! elimination from gut

constant Kguc=0.605 ! optimization

! Kguc (1/h) is the elimination rate constant with feces for the unabsorbed enrofloxacin.

! elimination from metabolism

constant Kf=0.0725 ! optimization; Kf (1/h) is the metabolism rate constant from enrofloxacin to ciprofloxacin.

! elimination from Renal excretion

constant Clreperkg=0.058 ! L/kg/h; renal clearance for enrofloxacin; optimization

Clre=Clreperkg*bw

! elimination from exchange between gill and water body

constant Kgw=0.061 ! 1/h

! drug degradation in water

constant Kde=12003.31 ! 1/h

! elimination for ciprofloxacin

constant Clmreperkg=116.14 ! L/kg/h; renal clearance for ciprofloxacin; optimization

Clmre=Clmreperkg*bw

! molecular weight of enrofloxacin and ciprofloxacin

constant MWENR=359.4, MWCIP=331.347 ! g/mol

! dose

constant perdoseiv=10, perdosepo=10 ! mg/kg bw

constant perdoseib=10 ! ppm in water for 2.5 h exposure, equal to mg/kg or mg/L; ([6](#_ENREF_6))

constant Vwater=40 ! L

doseib=perdoseib*Vwater !! enrofloxacin IBly given, mg

doseiv=perdoseiv*bw !! enrofloxacin IVly given, mg

dosepo=perdosepo*bw !! enrofloxacin POly given, mg

END ! INITIAL

DYNAMIC

ALGORITHM IALG = 2

NSTEPS NSTP = 10

MAXTERVAL MAXT = 1.0e9

MINTERVAL MINT = 1.0e-9

CINTERVAL CINT = 0.1

DERIVATIVE

!!!!!!!!!!!!!! code for enrofloxacin is listed below

!! multiple doses code

CONSTANT timepo=0.001 ! Length of oral gavage exposure (h)

constant timeiv=0.001 ! Length of IV exposure (h)

constant timeib=1 ! Length of immersion bath exposure (h); 0.5 h for 100 ppm IB; ([6](#_ENREF_6))

CONSTANT tinterval = 24 ! Varied dependent on the exposure (h)

CONSTANT Dstart = 0 ! Initiation day of the first dose (day)

CONSTANT Dstop = 1 ! Termination day of the last dose (day)

Tsim = TSTOP ! Tstop in hours

DS = Dstart*24 ! Initiation time point of oral gavage (h)

Doff = (Dstop - Dstart)*24 !

ExposureIB = PULSE(DS, Tsim, Doff)*PULSE(0,tinterval,timeib) ! pulse function to simulate the IB dosing time

ExposurePO = PULSE(DS, Tsim, Doff)*PULSE(0,tinterval,timepo) ! pulse function to simulate the IB dosing time

ExposureIV = PULSE(DS, Tsim, Doff)*PULSE(0,tinterval,timeiv) ! pulse function to simulate the IB dosing time

!! multiple PO dosing

Rdosepo=dosepo/timepo*ExposurePO

poin=integ(Rdosepo,0)

!! multiple IV dosing

Rdoseiv=doseiv/timeiv*ExposureIV

ivin=integ(Rdoseiv,0)

!! multiply IB dosing

Rdoseib=doseib/timeib*ExposureIB

ibin=integ(Rdoseib,0)

MASSIN=poin+ivin+ibin

!!!!!!!!!!!!!!!!!!!

! code for simulating the drug exchange between water and fish (gill)

raw=-KaIB*aw+Kgw*agi+Reli1+Reli3+Rdoseib-Rade ! mg/h

Rade=Kde*aw !mg/h

aw=integ(raw,0) !mg

Cwater=aw/Vwater !mg/L, equal to ug/ml

aucwa=integ(Cwater,0) !mg*h/L

MASSOUT=integ(Rade,0)+outbio

!!!!!!!!!!!!!!!!!!!

! code for simulating the enrofloxacin in the fish body

! gill

ragi=Qtot*(Cvb-Cgi/Pgi)-Kgw*agi !mg/h

agi=integ(ragi,0) !mg

Cgi=agi/Vgi !mg/L, equal to ug/ml

aucgi=integ(Cgi,0) !ug*h/ml; AUC

! Stomach contents

rastc=Rdosepo-Kst*astc !mg/h

astc=integ(rastc,0) !mg

! Intestinal contents

raic=Kst*astc-F*KaPO*aic-Reli1 !mg/h

Reli1=Kguc*aic*(1-F) !mg/h

aic=integ(raic,0) !mg

! gut

rag=Qg*(Cab-Cg/Pg) !mg/h

ag=integ(rag,0) !mg

Cg=ag/Vg !mg/L

aucgu=integ(Cg,0) !ug*h/ml; AUC

! liver

ral=F*KaPO*aic+Ql*Cab+Qg*Cg/Pg-(Ql+Qg)*Cl/Pl-Reli2 !mg/h

Reli2=Kf*al !mg/h

al=integ(ral,0) !mg

Cl=al/Vl !mg/L

outbio=integ(Reli2,0) !mg

aucli=integ(Cl,0) !ug*h/ml; AUC

! kidney

rak=Qk*(Cab-Ck/Pk)-Reli3 !mg/h

ak=integ(rak,0) !mg

Ck=ak/Vk !mg/L

aucki=integ(Ck,0) !ug*h/ml; AUC

Reli3=Clre*Ck/Pk !mg/h

out3=integ(Reli3,0) !mg

! muscle

ram=Qm*(Cab-Cm/Pm) !mg/h

am=integ(ram,0) !mg

Cm=am/Vm !mg/L

aucmu=integ(Cm,0) !ug*h/ml; AUC

! skin

ras=Qs*(Cab-Cs/Ps) !mg/h

as=integ(ras,0) !mg

Cs=as/Vs !mg/L

aucsk=integ(Cs,0) !ug*h/ml; AUC

! rest

rar=Qr*(Cab-Cr/Pr) !mg/h

ar=integ(rar,0) !mg

Cr=ar/Vr !mg/L

aucre=integ(Cr,0) !ug*h/ml; AUC

! Venous blood

ravb=Rdoseiv+KaIB*aw+Qr*Cr/Pr+Qs*Cs/Ps+Qm*Cm/Pm+Qk*Ck/Pk+(Ql+Qg)*Cl/Pl-Qtot*Cvb !mg/h

avb=integ(ravb,0) !mg

Cvb=avb/Vvb !mg/L

Cvp=avb/Vvp !mg/L

Cvs=avb/Vvs !mg/L

aucvb=integ(Cvb,0) !ug*h/ml; AUC

aucvp=integ(Cvp,0) !ug*h/ml; AUC

aucvs=integ(Cvs,0) !ug*h/ml; AUC

! Arterial blood

raab=Qtot*(Cgi/Pgi-Cab) !mg/h

aab=integ(raab,0) !mg

Cab=aab/Vab !mg/L

Cap=aab/Vap !mg/L

Cas=aab/Vas !mg/L

!!!!!!!!!!!!!!!!!!! check the mass balance for enrofloxacin

MBenr=MASSIN-MASSOUT-(aw+aab+avb+agi+ar+ag+as+am+ak+al+aic+astc) ! As time increases, the value should eventually approach zero.

!!!!!!!!!!!!!!!!!!!!!!!!!!!!!!!!!!!!!!!!!!!!!!!!!!!!!!!!!!!!!!!!!!!!!!!!!!!!!!!

! code for ciprofloxacin is listed below

! biotransform enrflocaxin to ciprofloxacin

Rbiotrans=Kf*al/MWENR*MWCIP !mg/L

! liver

raml=Rbiotrans+(Ql+Qg)*(Cmab-Cml/Pml) !mg/h

aml=integ(raml,0) !mg

Cml=aml/Vl !mg/L

aucMli=integ(Cml,0) ! AUC for CIP in the liver

MASSMin=integ(Rbiotrans,0)

! KIDNEY

ramk=Qk*(Cmab-Cmk/Pmk)-relim !mg/h

relim=Clmre*Cmk/Pmk !mg/h

amk=integ(ramk,0) !mg

Cmk=amk/Vk !mg/L

mout=integ(relim,0) !mmol

aucMki=integ(Cmk,0) ! AUC for CIP in the kidney

MASSMout=mout

! muscle

ramm=Qm*(Cmab-Cmm/Pmm) !mg/h

amm=integ(ramm,0) !mg

Cmm=amm/Vm !mg/L

aucMmu=integ(Cmm,0) ! AUC for CIP in the muscle

! skin

rams=Qs*(Cmab-Cms/Pms) !mg/h

ams=integ(rams,0) !mg

Cms=ams/Vs !mg/L

aucMsk=integ(Cms,0) ! AUC for CIP in the skin

! rest

ramr=Qr*(Cmab-Cmr/Pmr) !mg/h

amr=integ(ramr,0) !mg

Cmr=amr/Vmr !mg/L

aucMre=integ(Cmr,0) ! AUC for CIP in the rest compartment

! gill

ramgi=Qtot*(Cmvb-Cmgi/Pmgi) !mg/h

amgi=integ(ramgi,0) !mg

Cmgi=amgi/Vgi !mg/L

aucMgi=integ(Cmgi,0) ! AUC for CIP in the gills

! Venous blood

ramvb=Qr*Cmr/Pmr+Qs*Cms/Pms+Qm*Cmm/Pmm+Qk*Cmk/Pmk+(Ql+Qg)*Cml/Pml-Qtot*Cmvb !mg/h

amvb=integ(ramvb,0) !mg

Cmvb=amvb/Vvb !mg/L

Cmvp=amvb/Vvp !mg/L

Cmvs=amvb/Vvs !mg/L

aucMvp=integ(Cmvp,0) !AUC for CIP in the venous plasma

aucMvs=integ(Cmvs,0) !AUC for CIP in the venous serum

! Arterial blood

ramab=Qtot*(Cmgi/Pmgi-Cmab) !mg/h

amab=integ(ramab,0) !mg

Cmab=amab/Vab !mg/L

Cmap=amab/Vap !mg/L

Cmas=amab/Vas !mg/L

aucMap=integ(Cmap,0) !AUC for CIP in the arterial plasma

aucMas=integ(Cmas,0) !AUC for CIP in the arterial serum

!!!!!!!!!!!!!!!!!!! check the mass balance for ciprofloxacin

MBcip=MASSMin-MASSMout-(amab+amvb+amgi+amr+ams+amm+amk+aml) ! As time increases, the value should eventually approach zero.

END ! DERIVATIVE

CONSTANT TSTOP =100

! CHECK P

ppki=aucki/aucvb

ppmu=aucmu/aucvb

ppsk=aucsk/aucvb

ppgu=aucgu/aucvb

ppgi=aucgi/aucvb

ppre=aucre/aucvb

TERMT (T .GE. TSTOP, 'checked on communication interval: REACHED TSTOP')

END ! DYNAMIC

END ! PROGRAM

**2 M-type code file used to calculate the withdrawal intervals**

MRLmu=0.1; % ug/g, EMA 2002

MRLsk=0.1; % ug/g, EMA 2002

maxt=max(_t);

time=[0:4:maxt];

time=flipud(time);

nn=length(time);

one=ones(1,nn);

mu=one*MRLmu;

sk=one*MRLsk;

mu=flipud(mu);

sk=flipud(sk);

plot(time,mu,_t,__ctotalmu_th__);

plot(time,sk,_t,__ctotalsk_th__);

ta=flipud(_t);

cmutoa=flipud(__ctotalmu_th__);

csktoa=flipud(__ctotalsk_th__);

for b=1:numIts;

muCH=find(cmutoa(:,b)>MRLmu);

if isempty (muCH)

wtmuCH(b)=0;

else

firmu=muCH(1)-1;

wtmuCH(b)=ta(firmu);

end

skCH=find(csktoa(:,b)>MRLsk);

if isempty (skCH)

wtskCH(b)=0;

else

firsk=skCH(1)-1;

wtskCH(b)=ta(firsk);

end

end

plot(wtmuCH);

plot(wtskCH);

**3 Supplementary Tables**

**Table S1.** The distribution information of those influential parameters subjected to Monte Carlo analysis

| Parameters | unit | Average value | SD | Min | Max | Reference |
| --- | --- | --- | --- | --- | --- | --- |
| Q_ck_ | unitless | 0.056 | 0.0056 | 0.0504 | 0.0616 | ([3](#_ENREF_3)) |
| V_cm_ | unitless | 0.66 | 0.066 | 0.594 | 0.726 | ([3](#_ENREF_3)) |
| V_cl_ | unitless | 0.029 | 0.0029 | 0.0261 | 0.0319 | ([3](#_ENREF_3)) |
| P_l_ | unitless | 4.9 | 0.49 | 4.41 | 5.39 | ([8](#_ENREF_8)) |
| P_m_ | unitless | 2.83 | 0.283 | 2.547 | 3.113 | ([8](#_ENREF_8)) |
| P_mm_ | unitless | 1.6 | 0.16 | 1.44 | 1.76 | ([7](#_ENREF_7)) |
| F | % | 66.13 | 6.613 | 59.517 | 72.743 | ([9](#_ENREF_9)) |
| K_f_ | h^-1^ | 0.0725 | 0.02 | 0.0525 | 0.0925 | Optimization |

Note: The water temperature was also an influential parameter. However, multiple different water temperatures were used in the previous studies ([6](#_ENREF_6), [8-11](#_ENREF_8)); therefore, although unlisted in this table, the water temperature was also subjected to the Monte Carlo analysis. Its distribution was based on the previous studies ([6](#_ENREF_6), [8-11](#_ENREF_8)) and had a mean value, a standard deviation (SD), a lower bound (Mean - SD), and an upper bound (Mean + SD).

**Table S2.** Normalized sensitivity coefficients of all 48 parameters for the sensitivity analysis about the influence of model parameters on the AUCs of enrofloxacin (ENR)

| Model parameters **^a^** | AUC_plasma_ | AUC_serum_ | AUC_muscle_ | AUC_kidney_ | AUC_liver_ | AUC_skin_ | AUC_gut_ |
| --- | --- | --- | --- | --- | --- | --- | --- |
| ***Blood flow (Q_cx_)*** | | | | | |  |  |
| Q_cgi_ | 0.00E+00 | 0.00E+00 | 0.00E+00 | 0.00E+00 | 0.00E+00 | 0.00E+00 | 0.00E+00 |
| Q_ck_ | -1.81E-01 | -1.81E-01 | -1.72E-01 | 5.39E-01 | -1.61E-01 | -1.53E-01 | -1.73E-01 |
| Q_cm_ | -1.38E-03 | -1.38E-03 | 4.57E-02 | -1.71E-03 | -2.78E-03 | -1.50E-02 | -3.07E-03 |
| Q_cs_ | -2.61E-02 | -2.61E-02 | -3.05E-02 | -2.69E-02 | -2.76E-02 | 2.88E-01 | -2.99E-02 |
| Q_cg_ | -1.96E-03 | -1.96E-03 | -2.37E-03 | -2.03E-03 | 1.41E-02 | -4.73E-03 | 3.92E-02 |
| Q_cl_ | -3.33E-04 | -3.33E-04 | -3.43E-04 | -3.35E-04 | 2.39E-03 | -3.95E-04 | -3.42E-04 |
| ***Tissue weight (V_cx_)*** | | | | | |  |  |
| V_cgi_ | -1.26E-01 | -1.26E-01 | -1.43E-01 | -1.47E-01 | -1.33E-01 | -1.32E-01 | -1.43E-01 |
| V_ck_ | -1.18E-03 | -1.18E-03 | -1.24E-03 | -7.86E-03 | -1.14E-03 | -1.38E-03 | -1.23E-03 |
| V_cm_ | -3.11E-01 | -3.11E-01 | -3.72E-01 | -3.13E-01 | -2.97E-01 | -3.43E-01 | -3.21E-01 |
| V_cs_ | -8.85E-02 | -8.85E-02 | -8.57E-02 | -8.81E-02 | -7.98E-02 | -4.04E-01 | -8.61E-02 |
| V_cg_ | -6.67E-02 | -6.67E-02 | -6.92E-02 | -6.72E-02 | -9.63E-02 | -7.41E-02 | -1.10E-01 |
| V_cl_ | -7.13E-02 | -7.13E-02 | -6.86E-02 | -7.07E-02 | -1.24E-01 | -6.25E-02 | -6.89E-02 |
| V_cvb_ | -9.20E-03 | -9.20E-03 | -9.65E-03 | -9.29E-03 | -8.87E-03 | -1.09E-02 | -9.59E-03 |
| V_cab_ | -2.14E-03 | -2.14E-03 | -2.45E-03 | -2.36E-03 | -2.25E-03 | -2.78E-03 | -2.44E-03 |
| ***Partition coefficient for ENR (P_x_)*** | | | | | |  |  |
| P_gi_ | -1.27E-01 | -1.27E-01 | -1.44E-01 | -1.48E-01 | -1.34E-01 | -1.33E-01 | -1.44E-01 |
| P_l_ | -7.16E-02 | -7.16E-02 | -6.89E-02 | -7.10E-02 | 8.76E-01 | -6.28E-02 | -6.92E-02 |
| P_k_ | -1.37E-03 | -1.37E-03 | -1.44E-03 | 9.92E-01 | -1.32E-03 | -1.61E-03 | -1.43E-03 |
| P_m_ | -3.26E-01 | -3.26E-01 | 6.13E-01 | -3.29E-01 | -3.11E-01 | -3.61E-01 | -3.37E-01 |
| P_s_ | -9.08E-02 | -9.08E-02 | -8.81E-02 | -9.04E-02 | -8.20E-02 | 5.94E-01 | -8.85E-02 |
| P_g_ | -6.86E-02 | -6.86E-02 | -7.13E-02 | -6.92E-02 | -9.82E-02 | -7.64E-02 | 8.87E-01 |
| P_r_ | -4.74E-04 | -4.74E-04 | -4.97E-04 | -4.78E-04 | -4.56E-04 | -5.61E-04 | -4.94E-04 |
| ***Partition coefficient for CIP (P_mx_)*** | | | | | |  |  |
| P_mgi_ | 0.00E+00 | 0.00E+00 | 0.00E+00 | 0.00E+00 | 0.00E+00 | 0.00E+00 | 0.00E+00 |
| P_ml_ | 0.00E+00 | 0.00E+00 | 0.00E+00 | 0.00E+00 | 0.00E+00 | 0.00E+00 | 0.00E+00 |
| P_mk_ | 0.00E+00 | 0.00E+00 | 0.00E+00 | 0.00E+00 | 0.00E+00 | 0.00E+00 | 0.00E+00 |
| P_mm_ | 0.00E+00 | 0.00E+00 | 0.00E+00 | 0.00E+00 | 0.00E+00 | 0.00E+00 | 0.00E+00 |
| P_ms_ | 0.00E+00 | 0.00E+00 | 0.00E+00 | 0.00E+00 | 0.00E+00 | 0.00E+00 | 0.00E+00 |
| P_mg_ | 0.00E+00 | 0.00E+00 | 0.00E+00 | 0.00E+00 | 0.00E+00 | 0.00E+00 | 0.00E+00 |
| P_mr_ | 0.00E+00 | 0.00E+00 | 0.00E+00 | 0.00E+00 | 0.00E+00 | 0.00E+00 | 0.00E+00 |
| ***Parameters related to drug absorption*** | | | | | |  |  |
| K_st_ | 3.94E-03 | 3.94E-03 | 4.44E-03 | 4.03E-03 | 4.03E-03 | 6.42E-03 | 4.37E-03 |
| K_aPO_ | 9.13E-02 | 9.13E-02 | 9.07E-02 | 9.12E-02 | 1.47E-01 | 8.81E-02 | 9.08E-02 |
| F | 2.64E-01 | 2.64E-01 | 2.61E-01 | 2.63E-01 | 4.29E-01 | 2.51E-01 | 2.62E-01 |
| K_aIB_ | 6.12E-02 | 6.12E-02 | 6.12E-02 | 6.12E-02 | 5.67E-02 | 6.13E-02 | 6.12E-02 |
| ***Parameters related to drug elimination*** | | | | | |  |  |
| K_guc_ | -8.84E-02 | -8.84E-02 | -8.75E-02 | -8.83E-02 | -1.44E-01 | -8.34E-02 | -8.76E-02 |
| K_f_ | -6.14E-02 | -6.14E-02 | -5.82E-02 | -6.08E-02 | -1.10E-01 | -5.09E-02 | -5.86E-02 |
| Cl_reperkg_ | -6.90E-02 | -6.90E-02 | -6.55E-02 | -7.79E-01 | -6.11E-02 | -5.74E-02 | -6.59E-02 |
| K_gw_ | -1.05E-01 | -1.05E-01 | -1.19E-01 | -1.24E-01 | -1.11E-01 | -1.05E-01 | -1.20E-01 |
| K_de_ | -6.22E-02 | -6.22E-02 | -6.22E-02 | -6.22E-02 | -5.77E-02 | -6.24E-02 | -6.22E-02 |
| Cl_mreperkg_ | 0.00E+00 | 0.00E+00 | 0.00E+00 | 0.00E+00 | 0.00E+00 | 0.00E+00 | 0.00E+00 |
| ***The other parameters*** | | | | | |  |  |
| Temp | -6.68E-01 | -6.68E-01 | -4.60E-01 | 1.46E+00 | -5.03E-01 | 3.31E-01 | -4.82E-01 |
| bw | -7.92E-02 | -7.92E-02 | -7.93E-02 | -7.92E-02 | -7.48E-02 | -7.94E-02 | -7.93E-02 |
| pcv | 4.37E-01 | 0.00E+00 | 0.00E+00 | 0.00E+00 | 0.00E+00 | 0.00E+00 | 0.00E+00 |
| pre | -2.33E-11 | 1.25E+00 | -2.49E-11 | -2.30E-11 | -2.32E-11 | -3.65E-11 | -2.70E-11 |
| MWENR | 0.00E+00 | 0.00E+00 | 0.00E+00 | 0.00E+00 | 0.00E+00 | 0.00E+00 | 0.00E+00 |
| MWCIP | 0.00E+00 | 0.00E+00 | 0.00E+00 | 0.00E+00 | 0.00E+00 | 0.00E+00 | 0.00E+00 |
| perdoseiv | 4.40E+00 | 4.40E+00 | 4.40E+00 | 4.40E+00 | 4.36E+00 | 4.40E+00 | 4.40E+00 |
| perdoseib | 3.85E+00 | 3.85E+00 | 3.85E+00 | 3.85E+00 | 3.86E+00 | 3.85E+00 | 3.85E+00 |
| perdosepo | 3.67E+00 | 3.67E+00 | 3.67E+00 | 3.67E+00 | 3.76E+00 | 3.67E+00 | 3.67E+00 |
| Vwater | 1.43E+01 | 1.43E+01 | 1.43E+01 | 1.43E+01 | 1.44E+01 | 1.43E+01 | 1.43E+01 |

Note: **^a^** All parameters listed here can be found in the model code written in acslXtreme software.

All parameters whose values are marked in red are the influential ones.

**Table S3.** Normalized sensitivity coefficients of all 48 parameters for the sensitivity analysis about the influence of model parameters on the AUCs of ciprofloxacin (CIP)

| Model parameters **^a^** | AUC_plasma_ | AUC_serum_ | AUC_muscle_ | AUC_kidney_ | AUC_liver_ | AUC_skin_ |
| --- | --- | --- | --- | --- | --- | --- |
| ***Blood flow (Q_cx_)*** | | | | | |  |
| Q_cgi_ | 0.00E+00 | 0.00E+00 | 0.00E+00 | 0.00E+00 | 0.00E+00 | 0.00E+00 |
| Q_ck_ | -6.20E-01 | -6.20E-01 | -6.02E-01 | 3.81E-01 | -4.62E-01 | -6.06E-01 |
| Q_cm_ | -3.19E-02 | -3.19E-02 | 1.53E-02 | -3.21E-02 | -2.26E-02 | -3.53E-02 |
| Q_cs_ | -4.15E-02 | -4.15E-02 | -4.33E-02 | -4.16E-02 | -3.71E-02 | -3.84E-03 |
| Q_cg_ | 2.45E-02 | 2.45E-02 | 2.55E-02 | 2.45E-02 | -2.60E-01 | 2.53E-02 |
| Q_cl_ | 5.23E-03 | 5.23E-03 | 5.54E-03 | 5.25E-03 | -4.86E-02 | 5.47E-03 |
| ***Tissue weight (V_cx_)*** | | | | | |  |
| V_cgi_ | -1.46E-01 |  | -1.48E-01 | -1.49E-01 | -1.43E-01 | -1.48E-01 |
| V_ck_ | -8.59E-04 | -1.46E-01 | -8.71E-04 | -8.66E-04 | -9.56E-04 | -8.68E-04 |
| V_cm_ | -6.64E-01 | -8.59E-04 | -7.26E-01 | -6.64E-01 | -5.41E-01 | -6.72E-01 |
| V_cs_ | -8.37E-02 | -6.64E-01 | -8.19E-02 | -8.35E-02 | -8.21E-02 | -1.21E-01 |
| V_cg_ | -1.29E-01 | -8.37E-02 | -1.33E-01 | -1.29E-01 | -1.19E-01 | -1.33E-01 |
| V_cl_ | 8.58E-01 | -1.29E-01 | 8.58E-01 | 8.58E-01 | 8.59E-01 | 8.58E-01 |
| V_cvb_ | -2.97E-02 | 8.58E-01 | -3.06E-02 | -2.98E-02 | -2.28E-02 | -3.04E-02 |
| V_cab_ | -7.16E-03 | -2.97E-02 | -7.78E-03 | -7.58E-03 | -5.79E-03 | -7.74E-03 |
| ***Partition coefficient for ENR (P_x_)*** | | | | | |  |
| P_gi_ | -1.16E-01 | -1.16E-01 | -1.13E-01 | -1.16E-01 | -1.21E-01 | -1.14E-01 |
| P_l_ | 8.82E-01 | 8.82E-01 | 8.83E-01 | 8.82E-01 | 8.80E-01 | 8.82E-01 |
| P_k_ | -1.56E-03 | -1.56E-03 | -1.59E-03 | -1.56E-03 | -1.48E-03 | -1.58E-03 |
| P_m_ | -3.35E-01 | -3.35E-01 | -3.38E-01 | -3.35E-01 | -3.27E-01 | -3.37E-01 |
| P_s_ | -6.55E-02 | -6.55E-02 | -6.33E-02 | -6.54E-02 | -7.08E-02 | -6.38E-02 |
| P_g_ | -1.31E-01 | -1.31E-01 | -1.36E-01 | -1.32E-01 | -1.21E-01 | -1.35E-01 |
| P_r_ | -5.46E-04 | -5.46E-04 | -5.58E-04 | -5.47E-04 | -5.18E-04 | -5.55E-04 |
| ***Partition coefficient for CIP (P_mx_)*** | | | | | |  |
| P_mgi_ | -3.38E-02 | -3.38E-02 | -3.75E-02 | -3.64E-02 | -2.42E-02 | -3.73E-02 |
| P_ml_ | -2.44E-02 | -2.44E-02 | -2.54E-02 | -2.44E-02 | 9.78E-01 | -2.51E-02 |
| P_mk_ | -1.01E-09 | -9.59E-10 | -1.05E-09 | 1.00E+00 | -7.03E-10 | -1.02E-09 |
| P_mm_ | -3.84E-01 | -3.84E-01 | 5.55E-01 | -3.85E-01 | -2.55E-01 | -3.91E-01 |
| P_ms_ | -2.64E-02 | -2.64E-02 | -2.71E-02 | -2.65E-02 | -1.75E-02 | 9.34E-01 |
| P_mg_ | 0.00E+00 | 0.00E+00 | 0.00E+00 | 0.00E+00 | 0.00E+00 | 0.00E+00 |
| P_mr_ | -6.03E-03 | -6.03E-03 | -6.21E-03 | -6.04E-03 | -4.00E-03 | -6.17E-03 |
| ***Parameters related to drug absorption*** | | | | | |  |
| K_st_ | 9.89E-03 | 9.89E-03 | 1.07E-02 | 9.94E-03 | 8.01E-03 | 1.05E-02 |
| K_aPO_ | 1.60E-01 | 1.60E-01 | 1.61E-01 | 1.60E-01 | 1.56E-01 | 1.61E-01 |
| F | 4.57E-01 | 4.57E-01 | 4.61E-01 | 4.57E-01 | 4.48E-01 | 4.60E-01 |
| K_aIB_ | 5.56E-02 | 5.56E-02 | 5.54E-02 | 5.56E-02 | 5.59E-02 | 5.55E-02 |
| ***Parameters related to drug elimination*** | | | | | |  |
| K_guc_ | -1.52E-01 | -1.52E-01 | -1.53E-01 | -1.52E-01 | -1.50E-01 | -1.53E-01 |
| K_f_ | 9.02E-01 | 9.02E-01 | 9.04E-01 | 9.02E-01 | 8.98E-01 | 9.03E-01 |
| Cl_reperkg_ | -4.79E-02 | -4.79E-02 | -4.62E-02 | -4.78E-02 | -5.21E-02 | -4.66E-02 |
| K_gw_ | -8.85E-02 | -8.85E-02 | -8.55E-02 | -8.83E-02 | -9.56E-02 | -8.62E-02 |
| K_de_ | -5.66E-02 | -5.66E-02 | -5.65E-02 | -5.66E-02 | -5.70E-02 | -5.65E-02 |
| Cl_mreperkg_ | -9.74E-05 | -9.74E-05 | -9.46E-05 | -1.00E+00 | -6.40E-05 | -9.52E-05 |
| ***The other parameters*** | | | | | |  |
| Temp | -1.92E+00 | -1.92E+00 | -1.73E+00 | 9.71E-01 | -2.39E+00 | -1.77E+00 |
| bw | -7.38E-02 | -7.38E-02 | -7.36E-02 | -7.38E-02 | -7.41E-02 | -7.37E-02 |
| pcv | 4.37E-01 | 0.00E+00 | 0.00E+00 | 0.00E+00 | 0.00E+00 | 0.00E+00 |
| pre | -4.08E-11 | 1.25E+00 | -4.46E-11 | -4.40E-11 | -3.77E-11 | -4.64E-11 |
| MWENR | -1.00E+00 | -1.00E+00 | -1.00E+00 | -1.00E+00 | -1.00E+00 | -1.00E+00 |
| MWCIP | 1.00E+00 | 1.00E+00 | 1.00E+00 | 1.00E+00 | 1.00E+00 | 1.00E+00 |
| perdoseiv | 4.35E+00 | 4.35E+00 | 4.35E+00 | 4.35E+00 | 4.35E+00 | 4.35E+00 |
| perdoseib | 3.87E+00 | 3.87E+00 | 3.87E+00 | 3.87E+00 | 3.87E+00 | 3.87E+00 |
| perdosepo | 3.77E+00 | 3.77E+00 | 3.78E+00 | 3.77E+00 | 3.77E+00 | 3.78E+00 |
| Vwater | 1.44E+01 | 1.44E+01 | 1.44E+01 | 1.44E+01 | 1.44E+01 | 1.44E+01 |

Note: **^a^** All parameters listed here can be found in the model code written in acslXtreme software.

All parameters whose values are marked in red are the influential ones.

**4 Supplementary Figures**

| **a**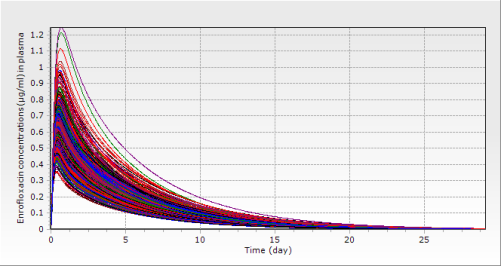 | **b**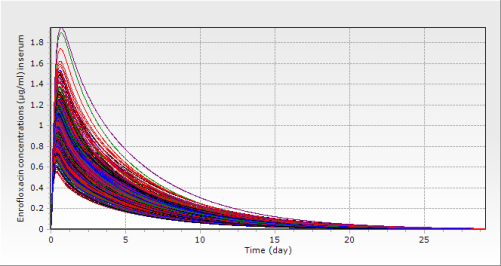 |
| --- | --- |
| **c**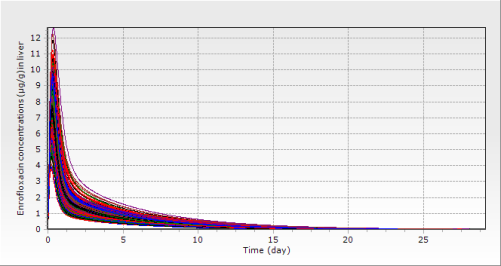 | **d**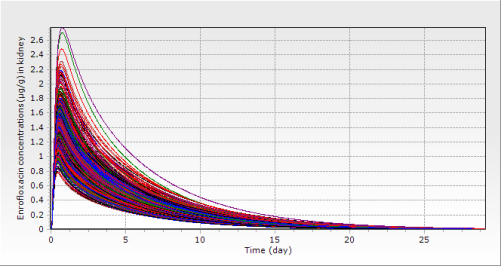 |
| **e**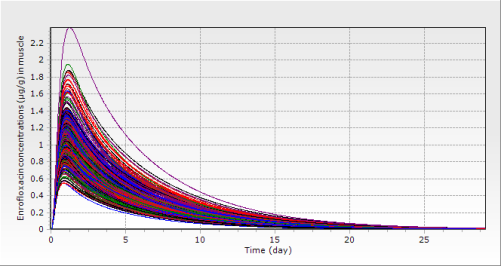 | **f**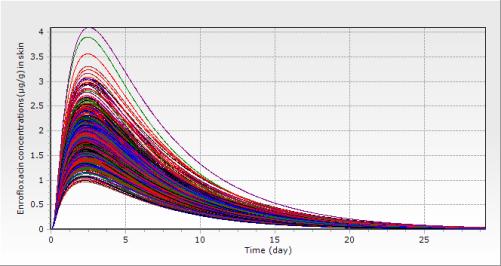 |

**Figure S1**. Predicted ENR concentrations (μg/ml or μg/g) after a single oral dose of ENR at 10 mg/kg BW in rainbow trout reared at 5 °C based on the Monte Carlo analysis with 500 iterations: a plasma, b serum, c liver, d kidney, e muscle, f skin

| **a** 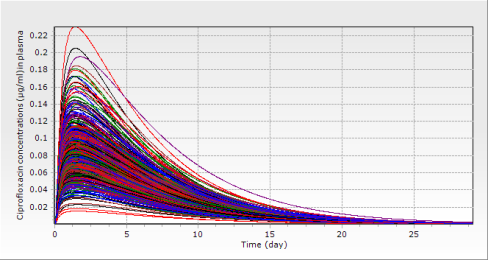 | **b** 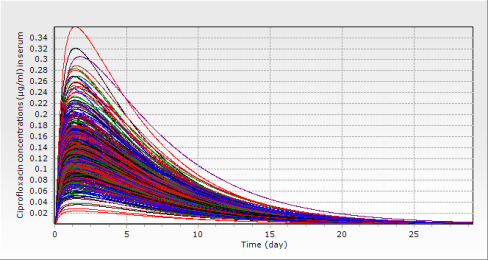 |
| --- | --- |
| **c 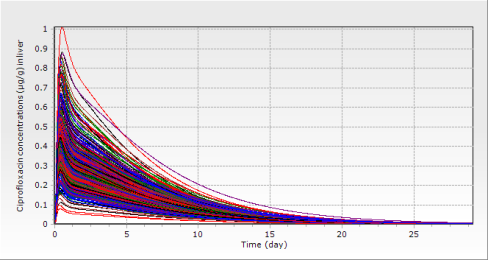** | **d** 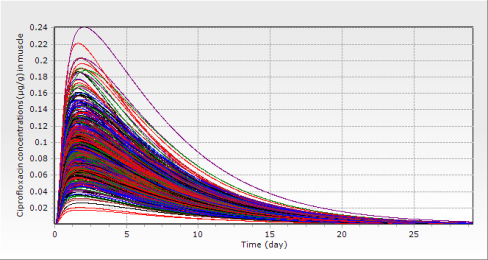 |
| **e** 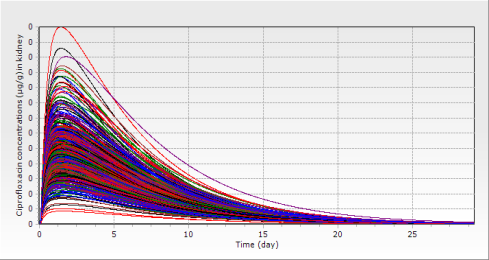 | **f 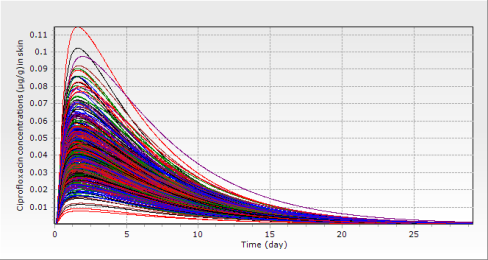** |

**Figure S2**. Predicted CIP concentrations (μg/ml or μg/g) after a single oral dose of ENR at 10 mg/kg BW in rainbow trout reared at 5 °C based on the Monte Carlo analysis with 500 iterations: a plasma, b serum, c liver, d muscle, e muscle, f skin

| **a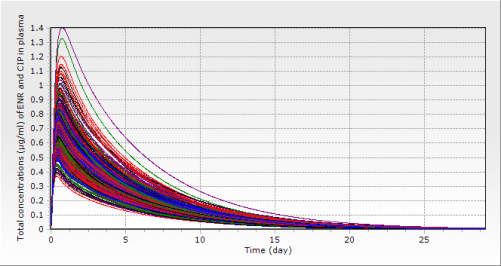** | **b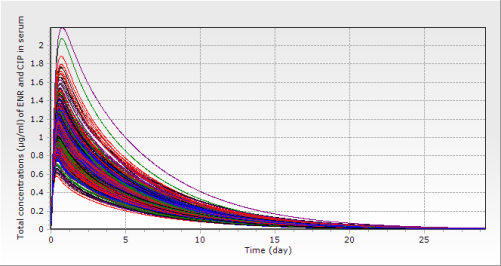** |
| --- | --- |
| **c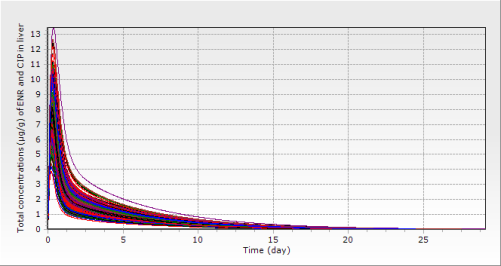** | **d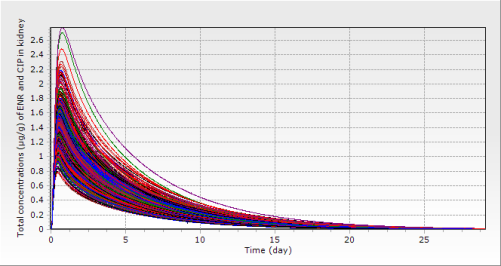** |
| **e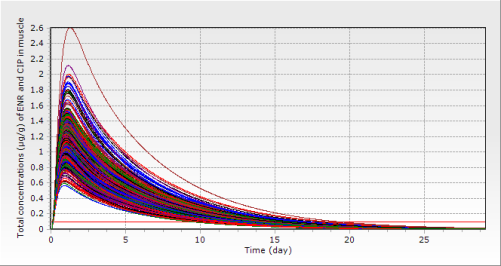** | **f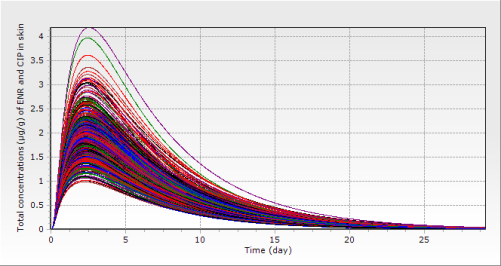** |

**Figure S3**. Predicted total concentrations (μg/ml or μg/g) of ENR and CIP after a single oral dose of ENR at 10 mg/kg BW in rainbow trout reared at 5 °C based on the Monte Carlo analysis with 500 iterations: a plasma, b serum, c liver, d kidney, e muscle, f skin

Note: The horizontal red straight line represents the MRL of ENR in the muscle (0.1 μg/g).

| **a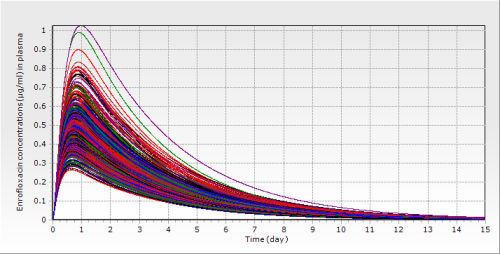** | **b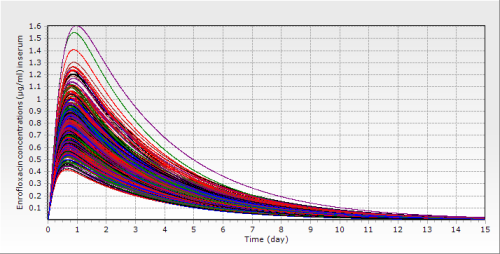** |
| --- | --- |
| **c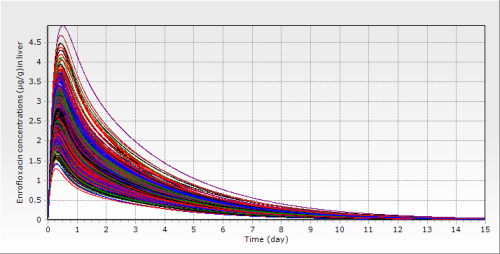** | **d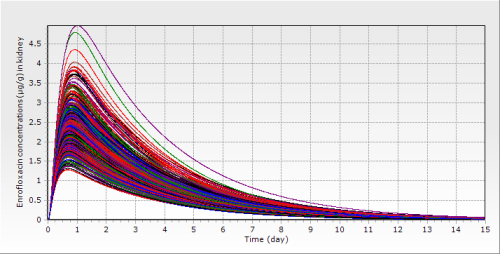** |
| **e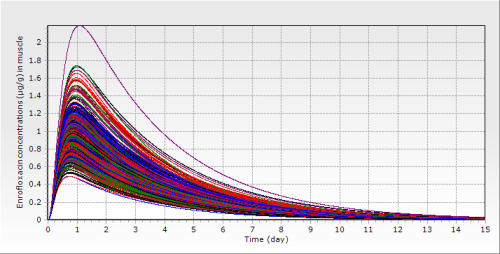** | **f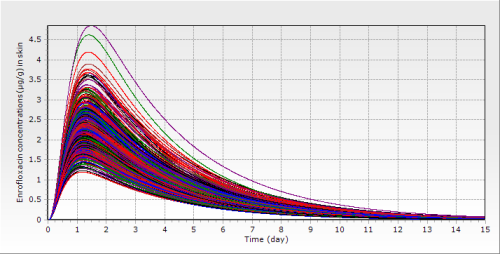** |

**Figure S4**. Predicted ENR concentrations (μg/ml or μg/g) after a single oral dose of ENR at 10 mg/kg BW in rainbow trout reared at 10 °C based on the Monte Carlo analysis with 500 iterations: a plasma, b serum, c liver, d kidney, e muscle, f skin

| **a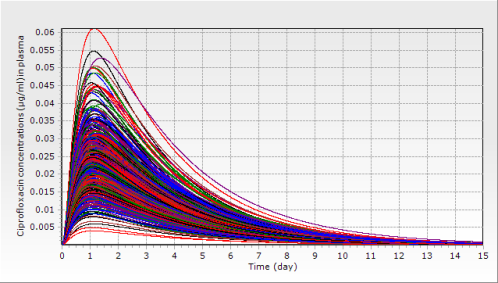** | **b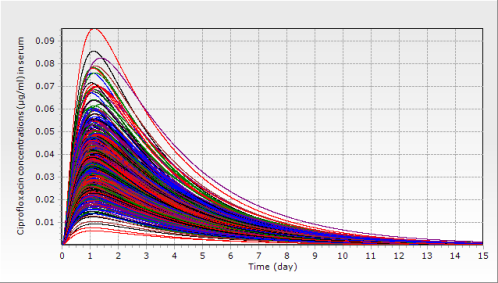** |
| --- | --- |
| **c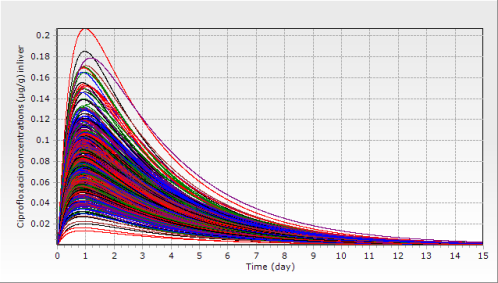** | **d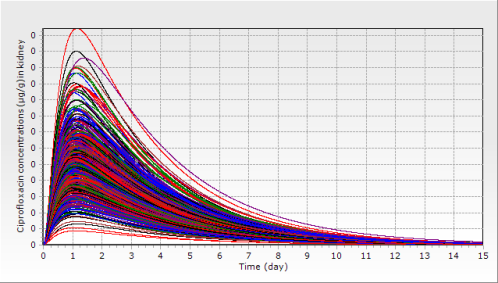** |
| **e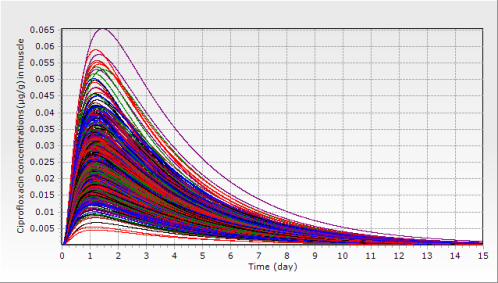** | **f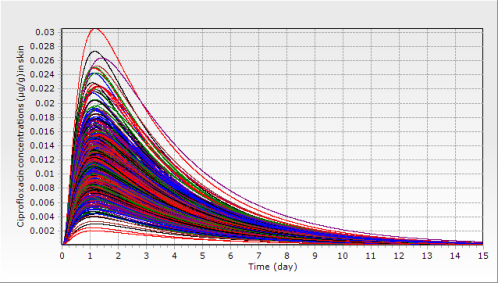** |

**Figure S5**. Predicted CIP concentrations (μg/ml or μg/g) after a single oral dose of ENR at 10 mg/kg BW in rainbow trout reared at 10 °C based on the Monte Carlo analysis with 500 iterations: a plasma, b serum, c liver, d kidney, e muscle, f skin

| **a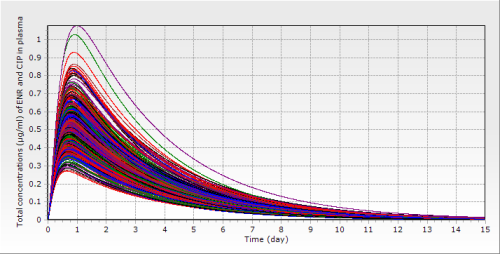** | **b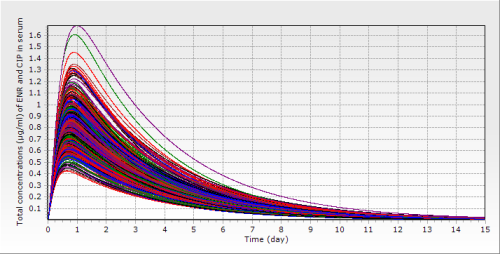** |
| --- | --- |
| **c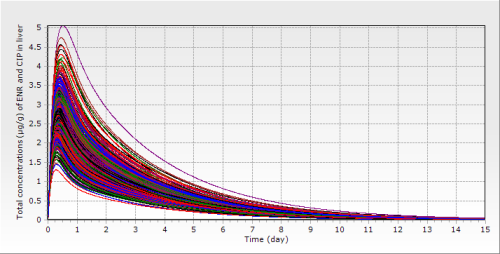** | **d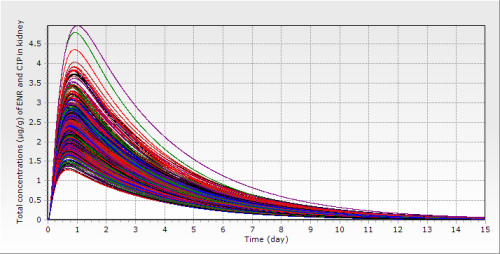** |
| **e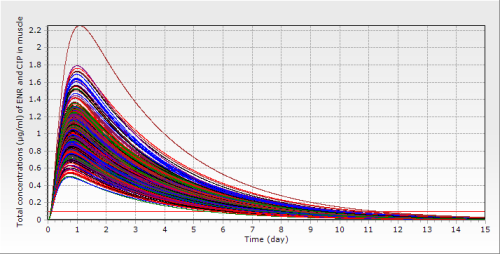** | **f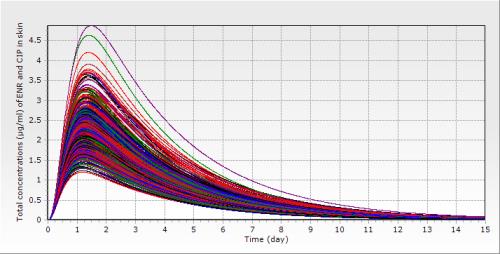** |

**Figure S6**. Predicted total concentrations (μg/ml or μg/g) of ENR and CIP after a single oral dose of ENR at 10 mg/kg BW in rainbow trout reared at 10 °C based on the Monte Carlo analysis with 500 iterations: a plasma, b serum, c liver, d kidney, e muscle, f skin

Note: The horizontal red straight line represents the MRL of ENR in the muscle (0.1 μg/g).

| **a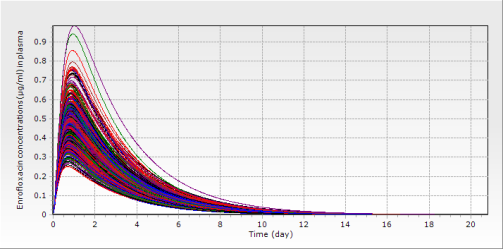** | **b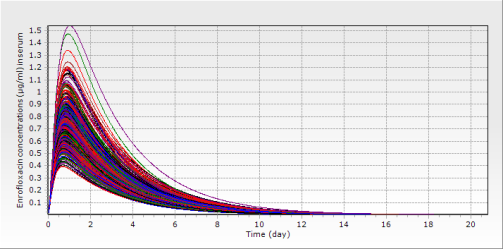** |
| --- | --- |
| **c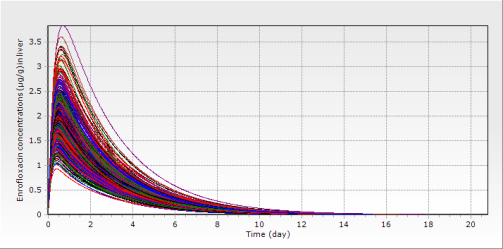** | **d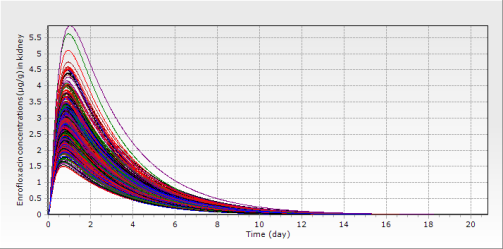** |
| **e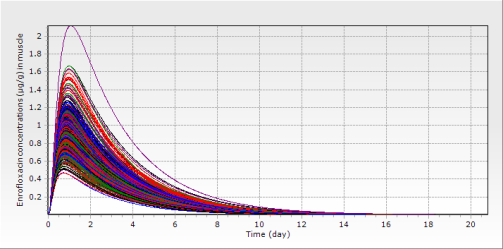** | **f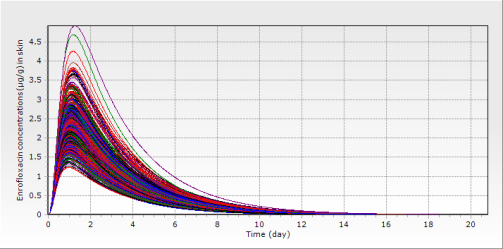** |

**Figure S7**. Predicted ENR concentrations (μg/ml or μg/g) after a single oral dose of ENR at 10 mg/kg BW in rainbow trout reared at 16 °C based on the Monte Carlo analysis with 500 iterations: a plasma, b serum, c liver, d kidney, e muscle, f skin

| **a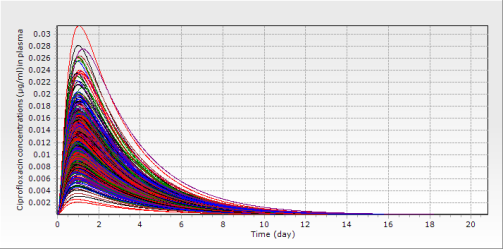** | **b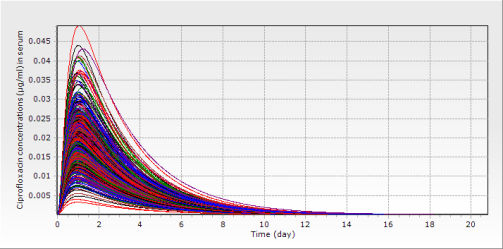** |
| --- | --- |
| **c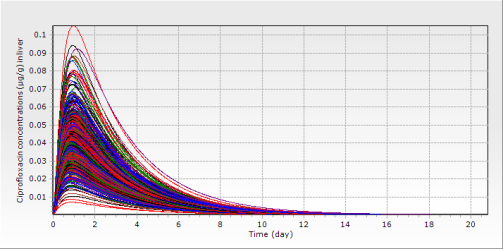** | **d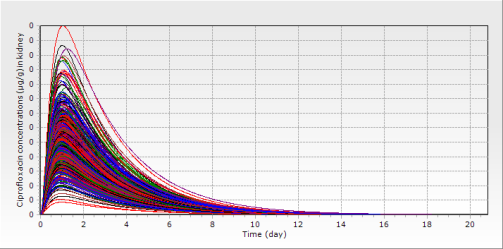** |
| **e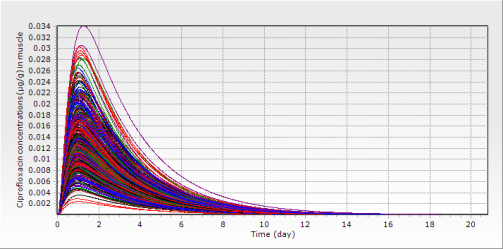** | **f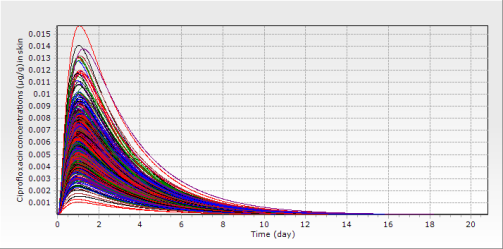** |

**Figure S8**. Predicted CIP concentrations (μg/ml or μg/g) after a single oral dose of ENR at 10 mg/kg BW in rainbow trout reared at 16 °C based on the Monte Carlo analysis with 500 iterations: a plasma, b serum, c liver, d kidney, e muscle, f skin

| **a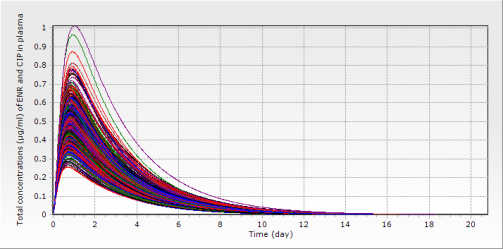** | **b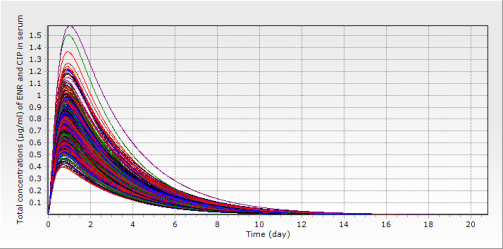** |
| --- | --- |
| **c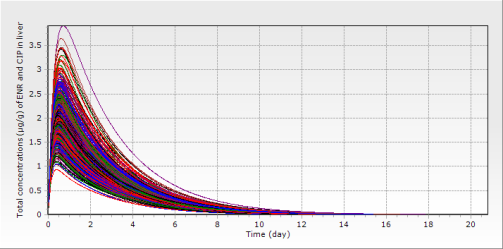** | **d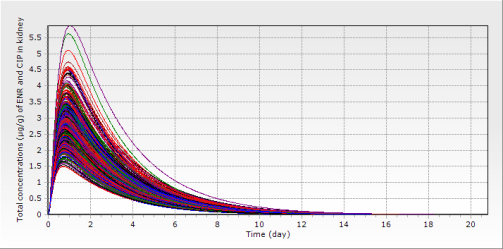** |
| **e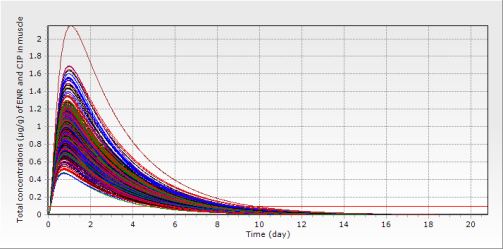** | **f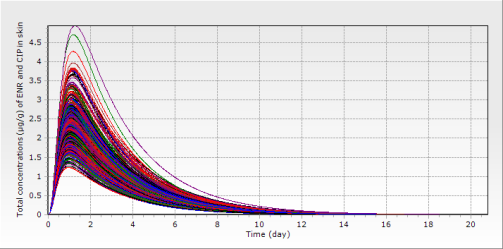** |

**Figure S9**. Predicted total concentrations (μg/ml or μg/g) of ENR and CIP after a single oral dose of ENR at 10 mg/kg BW in rainbow trout reared at 16 °C based on the Monte Carlo analysis with 500 iterations: a plasma, b serum, c liver, d kidney, e muscle, f skin

Note: The horizontal red straight line represents the MRL of ENR in the muscle (0.1 μg/g).

| **a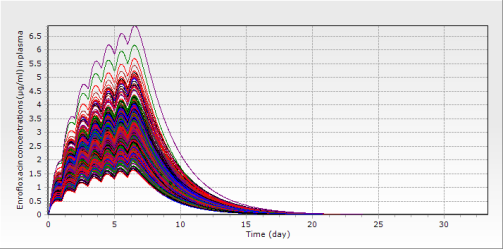** | **b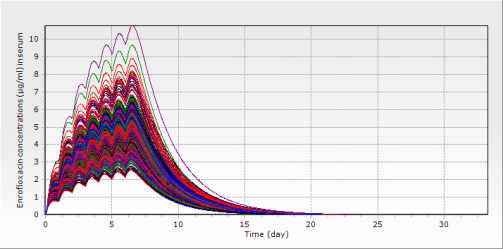** |
| --- | --- |
| **c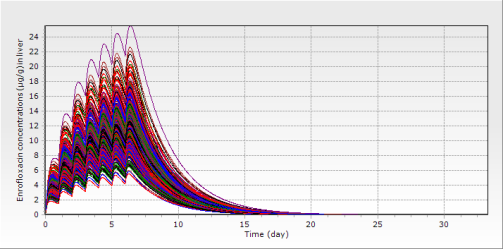** | **d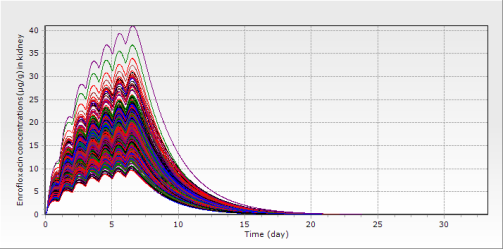** |
| **e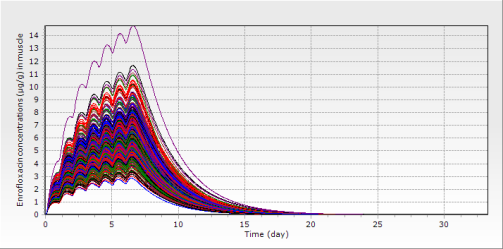** | **f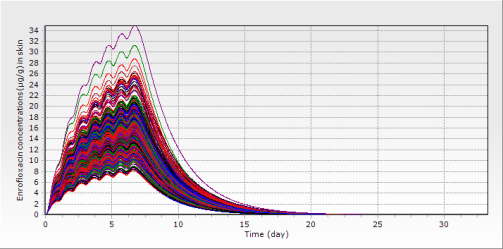** |

**Figure S10**. Predicted ENR concentrations (μg/ml or μg/g) after multiple oral doses of ENR at 20 mg/kg BW per day for seven consecutive days in rainbow trout reared at 16 °C based on the Monte Carlo analysis with 500 iterations: a plasma, b serum, c liver, d kidney, e muscle, f skin

| **a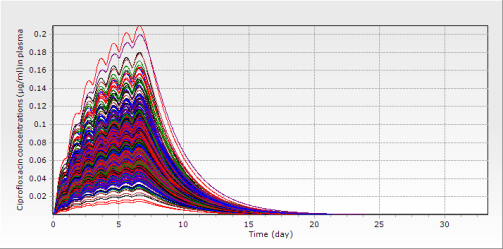** | **b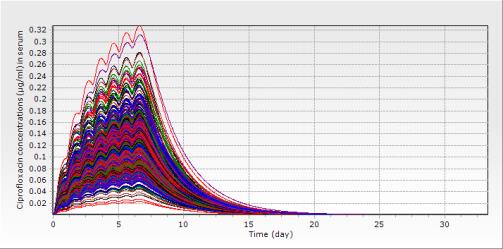** |
| --- | --- |
| **c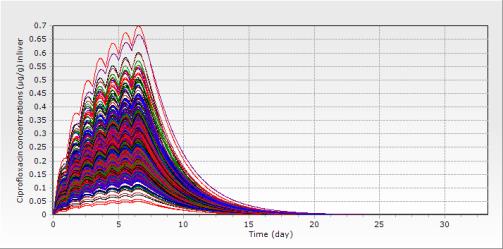** | **d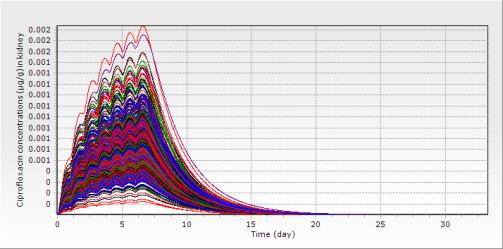** |
| **e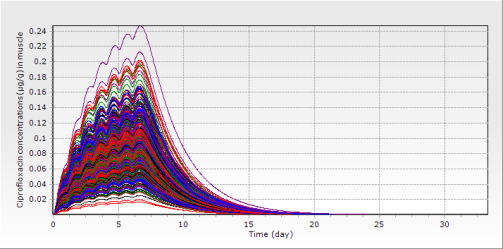** | **f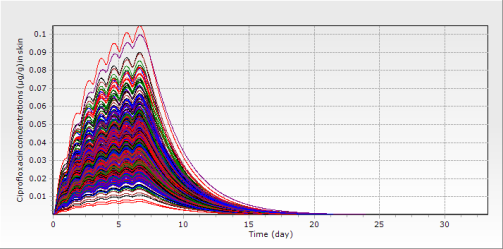** |

**Figure S11**. Predicted CIP concentrations (μg/ml or μg/g) after multiple oral doses of ENR at 20 mg/kg BW per day for seven consecutive days in rainbow trout reared at 16 °C based on the Monte Carlo analysis with 500 iterations: a plasma, b serum, c liver, d kidney, e muscle, f skin

| **a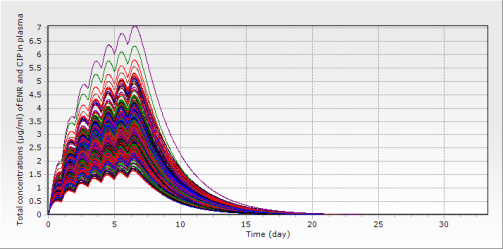** | **b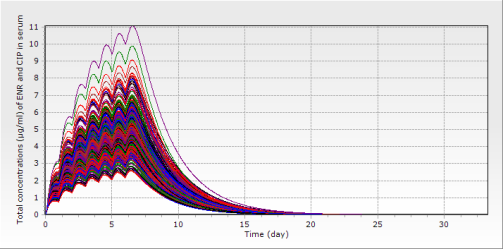** |
| --- | --- |
| **c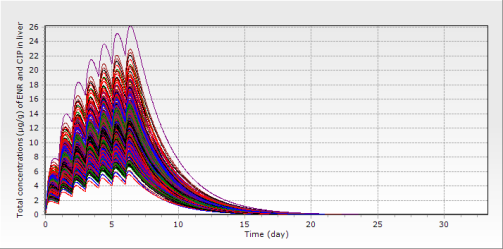** | **d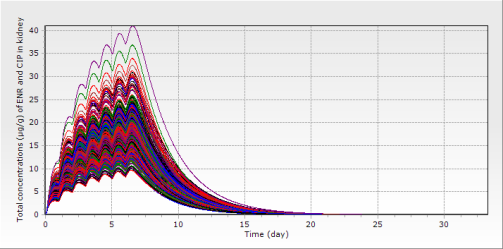** |
| **e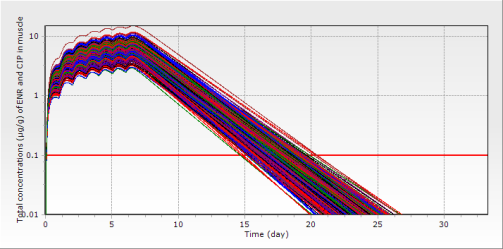** | **f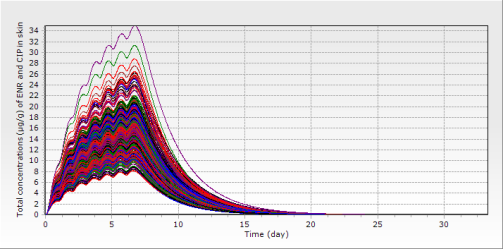** |

**Figure S12**. Predicted total concentrations (μg/ml or μg/g) of ENR and CIP after multiple oral doses of ENR at 20 mg/kg BW per day for seven consecutive days in rainbow trout reared at 16 °C based on the Monte Carlo analysis with 500 iterations: a plasma, b serum, c liver, d kidney, e muscle, f skin

Note: The horizontal red straight line represents the MRL of ENR in the muscle (0.1 μg/g).

| **a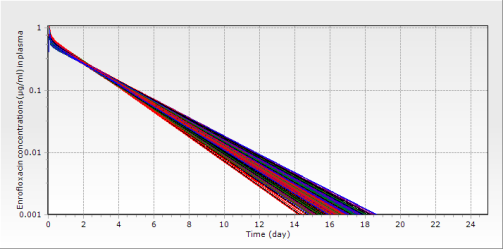** | **b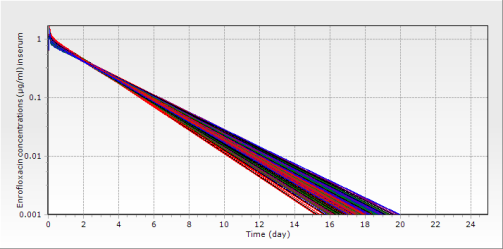** |
| --- | --- |
| **c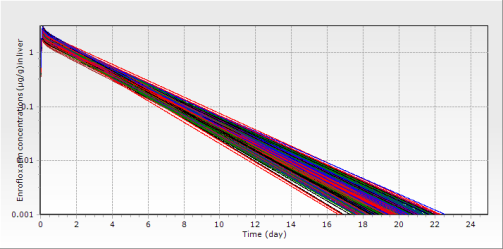** | **d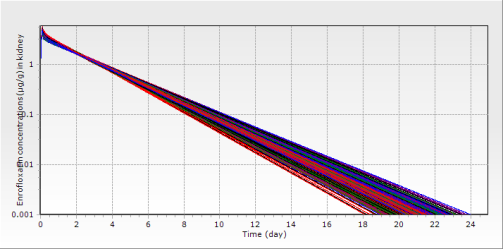** |
| **e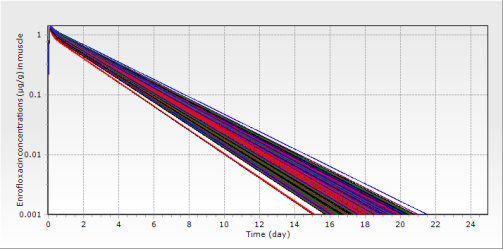** | **f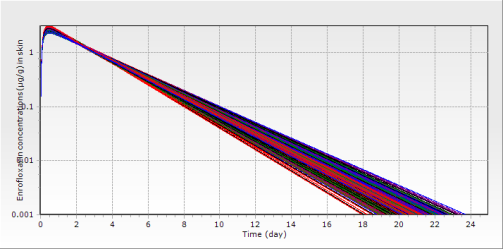** |

**Figure S13**. Predicted ENR concentrations (μg/ml or μg/g) after an immersion bath in water with ENR concentration at 20 ppm for 2.5 hours for rainbow trout reared at 16 °C based on the Monte Carlo analysis with 500 iterations: a plasma, b serum, c liver, d kidney, e muscle, f skin

| **a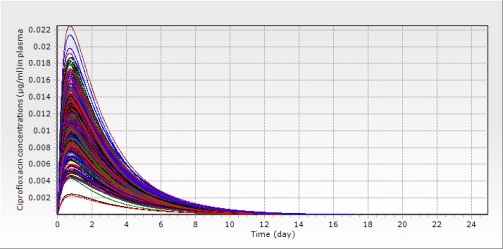** | **b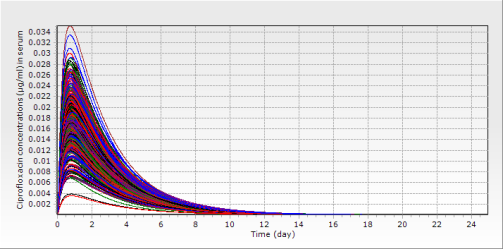** |
| --- | --- |
| **c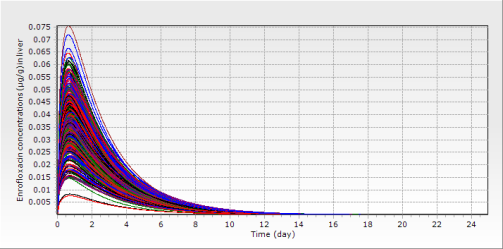** | **d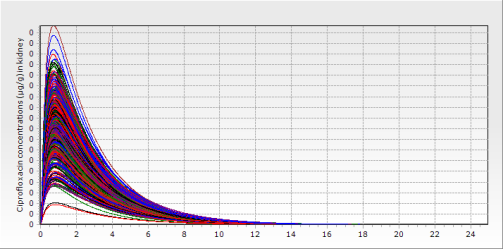** |
| **e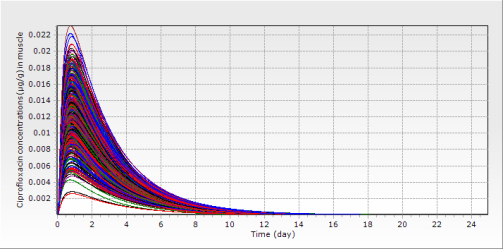** | **f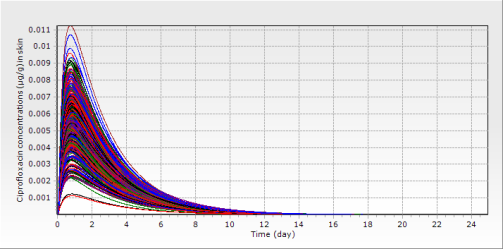** |

**Figure S14**. Predicted CIP concentrations (μg/ml or μg/g) after an immersion bath in water with ENR concentration at 20 ppm for 2.5 hours for rainbow trout reared at 16 °C based on the Monte Carlo analysis with 500 iterations: a plasma, b serum, c liver, d kidney, e muscle, f skin

| **a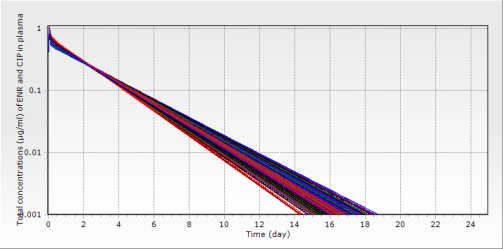** | **b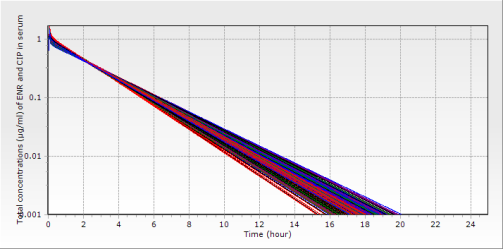** |
| --- | --- |
| **c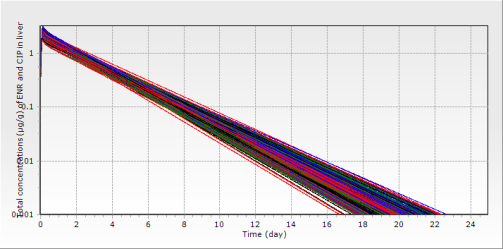** | **d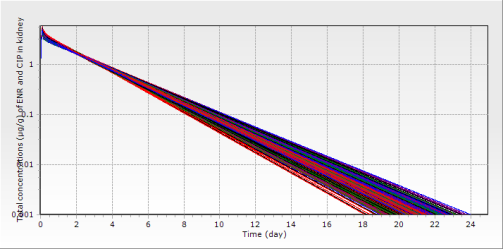** |
| **e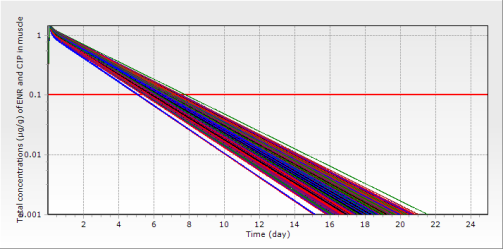** | **f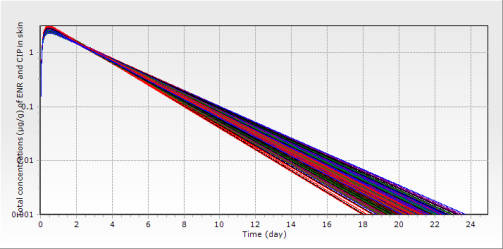** |

**Figure S15**. Predicted total concentrations (μg/ml or μg/g) of ENR and CIP after an immersion bath in water with ENR concentration at 20 ppm for 2.5 hours for rainbow trout reared at 16 °C based on the Monte Carlo analysis with 500 iterations: a plasma, b serum, c liver, d kidney, e muscle, f skin

Note: The horizontal red straight line represents the MRL of ENR in the muscle (0.1 μg/g).

| **a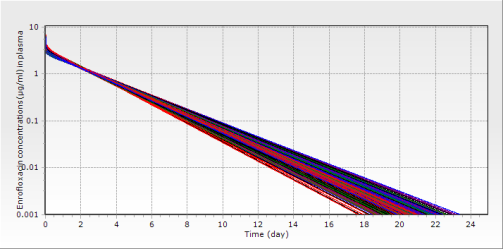** | **b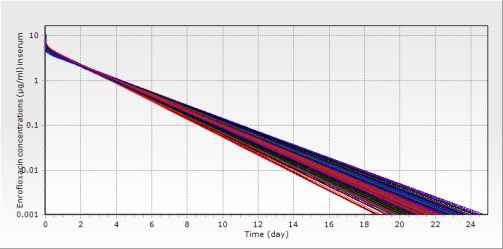** |
| --- | --- |
| **c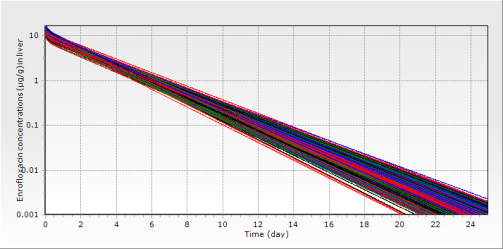** | **d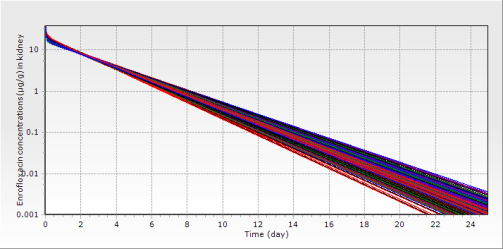** |
| **e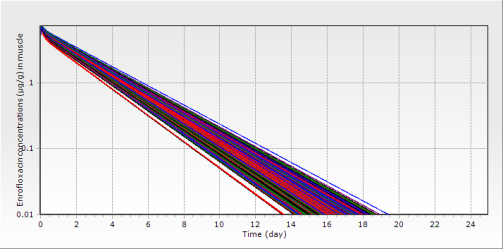** | **f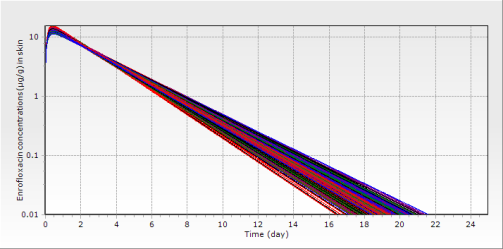** |

**Figure S16**. Predicted ENR concentrations (μg/ml or μg/g) after an immersion bath in water with ENR concentration at 100 ppm for 0.5 hours for rainbow trout reared at 16 °C based on the Monte Carlo analysis with 500 iterations: a plasma, b serum, c liver, d kidney, e muscle, f skin

| **a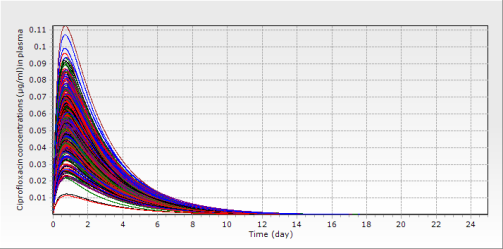** | **b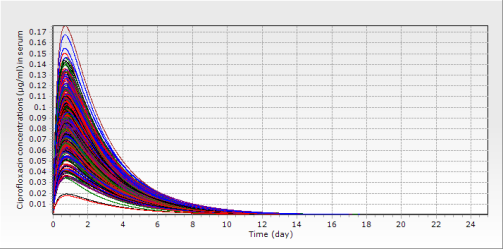** |
| --- | --- |
| **c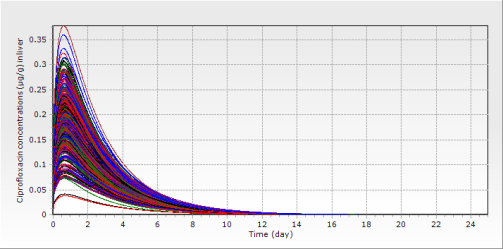** | **d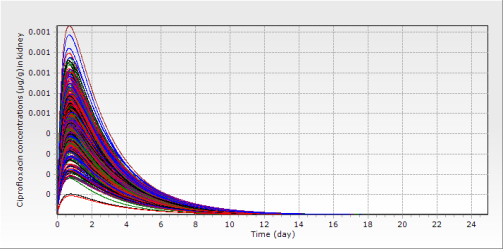** |
| **e** | **f** |

**Figure S17**. Predicted CIP concentrations (μg/ml or μg/g) after an immersion bath in water with ENR concentration at 100 ppm for 0.5 hours for rainbow trout reared at 16 °C based on the Monte Carlo analysis with 500 iterations: a plasma, b serum, c liver, d kidney, e muscle, f skin

| **a** | **b** |
| --- | --- |
| **c** | **d** |
| **e** | **f** |

**Figure S18**. Predicted total concentrations (μg/ml or μg/g) of ENR and CIP after an immersion bath in water with ENR concentration at 100 ppm for 0.5 hours for rainbow trout reared at 16 °C based on the Monte Carlo analysis with 500 iterations: a plasma, b serum, c liver, d kidney, e muscle, f skin

Note: The horizontal red straight line represents the MRL of ENR in the muscle (0.1 μg/g).

| **a** | **b** |
| --- | --- |

**Figure S19**. Distributions of withdrawal intervals in the muscle (a) and skin (b) after an immersion bath in water with ENR concentration of 20 ppm for 2.5 h based on the Monte Carlo analysis. Notes: the solid arrows indicated the 99th percentiles of the distribution at different water temperatures.

|  |  |
| --- | --- |

**Figure S20**. Distributions of withdrawal intervals in the muscle (a) and skin (b) after an immersion bath in water with ENR concentration of 10 ppm for 0.5 h based on the Monte Carlo analysis. Notes: the solid arrows indicated the 99th percentiles of the distribution at different water temperatures.

**Supplementary References**

1. Barron MG, Tarr BD, Hayton WL. Temperature-dependence of cardiac output and regional blood flow in rainbow trout, *Salmo gairdneri* Richardson. *J Fish Biol*. (1987) 31:735-744. doi: 10.1111/j.1095-8649.1987.tb05276.x

2. Yang F, Sun N, Sun YX, Shan Q, Zhao HY, Zeng DP, Zeng ZL. A physiologically based pharmacokinetics model for florfenicol in crucian carp and oral-to-intramuscular extrapolation. *J Vet Pharmacol Ther*. (2013) 36:192-200. doi: 10.1111/j.1365-2885.2012.01419.x

3. Abbas R, Hayton WL. A physiologically based pharmacokinetic and pharmacodynamic model for paraoxon in rainbow trout. *Toxicol Appl Pharmacol*. (1997) 145:192-201. doi: 10.1006/taap.1997.8168

4. Xu N, Li M, Chou WC, Lin Z. A physiologically based pharmacokinetic model of doxycycline for predicting tissue residues and withdrawal intervals in grass carp (*Ctenopharyngodon idella*). *Food Chem Toxicol*. (2020) 137:111127. doi: 10.1016/j.fct.2020.111127

5. Gallo JM, Lam FC, Perrier DG. Area method for the estimation of partition coefficients for physiological pharmacokinetic models. *J Pharmacokinet Biopharm*. (1987) 15:271-80. doi: 10.1007/BF01066322

6. Urzua N, Messina MJ, Prieto G, Luders C, Errecalde C. Pharmacokinetics and tissue disposition of enrofloxacin in rainbow trout after different routes of administration. *Xenobiotica*. (2020) 50:1236-1241. doi: 10.1080/00498254.2020.1747119

7. Sadiq MW, Nielsen EI, Khachman D, Conil JM, Georges B, Houin G, Laffont CM, Karlsson MO, Friberg LE. A whole-body physiologically based pharmacokinetic (WB-PBPK) model of ciprofloxacin: a step towards predicting bacterial killing at sites of infection. *J Pharmacokinet Pharmacodyn*. (2017) 44:69-79. doi: 10.1007/s10928-016-9486-9

8. Kyuchukova R, Milanova A, Pavlov A, Lashev L. Comparison of plasma and tissue disposition of enrofloxacin in rainbow trout (*Oncorhynchus mykiss*) and common carp (*Cyprinus carpio*) after a single oral administration. *Food Addit Contam Part A Chem Anal Control Expo Risk Assess*. (2015) 32:35-9. doi: 10.1080/19440049.2014.983998

9. Liang J, Li J, Zhao F, Liu P, Chang Z. Pharmacokinetics and tissue behavior of enrofloxacin and its metabolite ciprofloxacin in turbot *Scophthalmus maximus* at two water temperatures. *Chin J Oceanol Limnol*. (2012) 30:644-653. doi: 10.1007/s00343-012-1228-2

10. Bowser PR, Wooster GA, St Leger J, Babish JG. Pharmacokinetics of enrofloxacin in fingerling rainbow trout (*Oncorhynchus mykiss*). *J Vet Pharmacol Ther*. (1992) 15:62-71. doi: 10.1111/j.1365-2885.1992.tb00987.x

11. Pan H. Effect of water velocity, temperature and light intensity on pharmacokinetics of enrofloxacin in rainbow, Shanghai Ocean University, Shanghai Ocean University, Shanghai, 2017.
